# Research publications of Australia’s natural history museums, 1981–2020: Enduring relevance in a changing world

**DOI:** 10.1371/journal.pone.0287659

**Published:** 2023-06-23

**Authors:** Tayla A. Green, Pat A. Hutchings, Fiona R. Scarff, James R. Tweedley, Michael C. Calver

**Affiliations:** 1 Environmental and Conservation Sciences, Murdoch University, Murdoch, Western Australia, Australia; 2 Australian Museum Research Institute, Australian Museum, Sydney, NSW, Australia; 3 Department of Biological Sciences, Macquarie University, North Ryde, Australia; Instituto Tecnologico Autonomo de Mexico, MEXICO

## Abstract

As a case study of the responses of natural history museums to changing scientific and funding environments, we analysed research publications of Australia’s Natural History Museums (ANHMs) 1981–2020. Using Scopus, 9,923 relevant documents 1981–2020 were identified, mainly research papers but with a growing proportion of reviews. The number of documents published increased over tenfold from 39 (1981) to 553 (2020), likely driven by collaborations (rising from 28.5% of documents 1981–1985 to 87.2% of documents 2016–2020), contributions from retired staff, and volunteer support. The mean length of documents (pages) ranged from a low of 15.3 in 2001–2005 to a high of 17.4 in 1991–1995, but this statistically significant result was trivial in practical terms. The sources (i.e., journals, book titles, conference proceedings) in which ANHM authors published changed over time, with growing proportions of publications in journals covering molecular ecology/phylogenetics and biological conservation. We identified the major areas of study canvassed within the corpus of publications by developing structural topic models based on patterns of word use in document titles, abstracts and keyword lists. The topics discovered included study subjects traditional for natural history museums (new taxa, phylogeny, systematics, animal morphology, palaeontology, minerals), new directions (molecular genetics, ecology, biological conservation) and marine biology (probably reflecting Australia’s large coastline). Most citations came from Australia, USA and UK, although in 2016–2020 only 27.9% of citing documents included an Australian author. Growth in numbers of documents and collaborations, as well as use of documents internationally over a period of great change in scientific and funding environments, indicate an enduring legacy of ANHM research, grounded on the intrinsic value of the collections.

## Introduction

Globally, natural history museums contribute to four key areas: collecting, preserving, displaying, and researching [1]. In practical experience, research is often asked to justify itself [2], struggling to maintain relevance in the face of three worrying issues: tight funding, relevance of actual collections in the face of digitisation and the separation of research from display/education functions [3–5]. Additionally, since 1950 important new scientific disciplines relevant to natural history museum collections developed and matured: molecular biology [6–8], conservation biology [9, 10], restoration ecology [11, 12], and environmental science [13, 14]. While all have antecedents dating back well beyond the late 20^th^ century, they only matured recently. They operate in the context of sophisticated computer technology, including a push to digitise collections [15–18], as well as an increasing emphasis on databasing collections and collaborating in online resources such as the Atlas of Living Australia (https://www.ala.org.au).

Australia’s Natural History Museums (hereafter ANHMs) are a valuable case study of how the research outputs of natural history museums have responded to the challenges of maintaining relevance in the face of budgetary constraints, emerging new disciplines in science, and digitisation. Australia’s museum collections are a repository of Australia’s unique biodiversity [19–21], revealing changes in species distribution and abundance in natural communities, the forces shaping those communities, and the effects of modern landscape management, as well as fundamental biological data [22]. The collections also highlight the structure and diversity of Australia’s geology and physical environment [23, 24], as well as the history and culture of First Nations Australians [25, 26]. All these topics can be considered locally or regionally [27, 28], or globally [29–31].

In this study we use bibliometric tools to profile the publications of ANHMs 1981–2020. This date range covers the emergence of new areas of scientific inquiry relevant to museum research, the rise of digital technology and its application to museum collections and research, and a period of changing funding and social responsibility for museums. We define natural history broadly to include animals and their biotic and physical environments, explicitly including anthropological research because of humans’ role in ecosystems (all years of this study fall within the Anthropocene, [22]), and the earth and planetary sciences as studies of the physical environment. Herbaria in Australia are commonly separated from ANHMs so we excluded them, although we do include botanical documents published with at least one author affiliated with an ANHM, regarding plants as relevant to the biotic environment of animals.

Questions addressed include:

- What has been the productivity of ANHMs over time in respect to the number and type of peer-reviewed outputs published?
- What subjects were covered? Have there been changes over time?
- What are the nationalities and institutional affiliations of authors co-publishing with ANHM staff and affiliates? Have there been changes over time?
- Who cites ANHM research, based on the nationality and institutional affiliations of citing authors? Have there been changes over time?

These questions are most relevant to two of the five categories of museum-based research proposed by Sigfúsdóttir [5]: the scholar-curator model and the laboratory model. These share attention to traditional academic values, especially the importance of peer-reviewed written publication. We concentrate on them because of their suitability to bibliometric inquiry but do not imply any superiority to the other categories of practice-based research (linked to daily tasks and often with non-traditional outputs), co-researching with communities (empowering public knowledge or experience) and museological research (the study of museums and their functions).

## Materials and methods

### Ethics statement

No research ethics permits were required for this research.

### Australia’s natural history museums

This study considers eight museums, including any affiliated branch museums: Australian Museum, Museum and Art Gallery of the Northern Territory, Museum Victoria, National Museum of Australia, Queensland Museum, South Australian Museum, Tasmanian Museum and Art Gallery, and Western Australian Museum. In addition to seven State or Territory museums (the Australian Museum is actually the state museum for New South Wales), we also included the National Museum of Australia because its research includes a natural history component [32]. All mainland State museums exist via State legislation and follow relevant Australian Commonwealth regulations and guidelines, while the National Museum of Australia was established by Australian Commonwealth legislation (S1 Table). The exclusion of other Australian museums or the independent state herbaria is purely a matter of classification and does not imply any lack of significance in the research undertaken by staff at those institutions.

With the exception of the Museum and Art Gallery of the Northern Territory and the National Museum of Australia, all of the museums assessed were founded in the 19^th^ Century. Their most recent Annual Reports indicate a commitment to research, often with an explicit link to the value of their collections (S1 Table).

### Choice of database and search strategies

There are three widely used databases suitable for locating museums’ research publications: Scopus, Web of Science (WoS), and the Web of Science Core Collection (WoSCC), with a further option provided by the search engine Google Scholar (GS). Each has potential advantages and disadvantages [33–36]. We chose Scopus because it: has a comprehensive institutional affiliation feature, covers the period 1981–2020, lists a range of research outputs (sources in Scopus language), provides extensive bibliometric data, and has output replicable by anyone with a Scopus subscription following specified search strings [37–39]. Scopus refers to all records in the database as documents, which is the term we use to refer collectively to all items identified in searches. Relevant documents for all museums were identified using the ‘Advanced document search’ feature in Scopus on 22^nd^ April 2022, following the detailed protocol described in S1 File and using the affiliation codes in S2 Table. The final cleaned data file is included as S3 Table.

### Research outputs of the ANHMs

Annual numbers of documents attributed by Scopus to ANHMs were plotted as an indication of the volume of research published over time. As a check for possible bias in Scopus retrieval of pre-1996 documents in spite of the claims to have extended literature coverage back to 1970, we also retrieved data from WoSCC to see if the distribution across years was similar to Scopus. Affiliation searches in WoSCC found the names: Australian Museum, Museum Victoria, Queensland Museum, South Australian Museum and Western Australian Museum. We located documents from the other three museums by searching for a known document from each museum, copying the organisation string in the corresponding address, and then using that as a search term.

For convenience, descriptive data on documents were organised into eight groups, each comprising five years: 1981–1985, 1986–1990 and so on up to 2016–2020. Descriptive data presented include:

- Mean lengths of documents, calculated from the end page and start page entries in Scopus, using the formula (end page–start page) + 1. If end and start pages were unavailable, we located these documents online to determine the number of pages. Documents available as websites only were saved to pdf and the number of pages then counted.
- The Scopus classification of documents as: article (research paper), review, book chapter, conference paper, note (research note), letter, short survey, editorial, erratum and book.
- The percentage of documents published in Open Access (OA), further classified into: Gold (published by a dedicated OA publisher), Hybrid Gold (published in a subscription journal where authors may choose to publish OA), Bronze (the publisher has chosen to provide temporary or permanent free access), and Green (the author’s version of an accepted document deposited in a repository). For context, we also provide data on the percentage OA documents for Australia’s scientific output of 1,747,456 documents as a whole for the five year blocks beginning 1996–2000, using aggregated Scopus data from the SCImago Journal and Country Rank site (https://www.scimagojr.com/countrysearch.php?country=AU).
- The mean and median number of authors on documents, as well as the total number of unique authors/year group.
- The proportion of documents with an ANHM first author.

More detailed analyses were conducted on collaborations in authorship and subject areas covered.

#### Collaboration analysis: Who co-publishes with ANHM researchers?

We assessed collaboration within each of the year groups described above. From the author addresses recorded for each document retrieved, we tabulated for each year group the number of documents involving a co-author from (i) another Australian museum, (ii) an Australian university, (iii) any other Australian address (e.g., government departments, private research institutes or businesses, private individuals), (iv) an international museum, (v) an international university (including museums coming under the umbrella of a university), and (vi) any other international address (e.g., government departments, private research institutes or businesses, private individuals). Where an individual author gave two addresses, we included only the first mentioned. We also noted for each year group the top five nations contributing international co-authors, as well as mapping the data for all nations. For context, we also provide data on the percentage international collaboration (i.e., the percentage of documents with at least one non–Australian author) for Australia’s scientific output of 1,747,456 documents as a whole for the five year blocks beginning 1996–2000, using aggregated Scopus data from the SCImago Journal and Country Rank site (https://www.scimagojr.com/countrysearch.php?country=AU).

#### Subject areas covered

We predicted that between 1981 and 2020 there would be increases relative to other areas in publications covering:

- Genetics and molecular biology, reflecting the development and application of new molecular techniques.
- Biological conservation, ecology and environmental science, reflecting growing interest in these disciplines.
- Computer science, reflecting the push to digitise collections.

Some areas would need to decrease to balance these changes, so we predicted that these would likely occur in traditional core areas of ANHM work such as taxonomic descriptions (including anatomy and palaeontology) and rocks and minerals. We assessed subject areas by examining the sources in which ANHM authors published, supplemented by structural topic modelling of the text contained in document titles, keywords and abstracts, to identify prominent topics.

For the source analysis, we first located all cases where a journal had changed its name regarding all papers as belonging to the new name. A data matrix of the number of documents published in each of the 1,260 sources was constructed (S4 Table lists all sources, together with 2020 Scopus journal percentile rankings if available). The number of documents published varied annually, so data were standardised using totals from each year, creating percentage composition data (0–100), and then square-root transformed. A Bray-Curtis resemblance matrix was created from the transformed data and analysed using one-way Analysis of Similarities (ANOSIM; [40], to determine any significant difference (*p* < 0.05) in the sources museum authors published in over the year groups. The magnitude of the *R*-statistic calculated by ANOSIM ranges from 1, when all samples within each group are more similar to each other than to any of the samples from other groups, down to ~0, when the average similarities among and within groups do not differ. A second analysis using the RELATE procedure [40] tested for seriation over the forty years (*p* < 0.05), i.e., increasing separation of successive years, using the above Bray-Curtis resemblance matrix. The test statistic rho reflects the strength of the seriation from ~0 (little correlation) to ~1 (near-perfect correlation). Annual publication data were visualised using non-metric multidimensional scaling (nMDS) with a trajectory added to the ordination showing the linear progression of samples (years) over time.

The pattern of change in the relative counts for publications in individual sources across the forty years was examined using ‘coherent species curves’ [41]. This routine identifies, via cluster analysis, the groups of sources whose patterns of publications over the years are indistinguishable within a group but statistically significant between groups (*p* < 0.05), when tested using type 3 SIMPROF permutation procedure. Note that, as conducting this analysis on the full suite of 1,260 sources would only add random noise to the similarities, only the 50 sources making the largest contributions to the total number of documents in one or more of the forty years were used. Some of the resulting ‘coherent species groups’ were visualised using line plots of the percentage contribution of documents published in each year to qualitatively assess our predictions regarding subject areas growing relative to others. All analyses were conducted using PRIMER V7 [42].

Source analysis was complemented with text mining of the document titles, author keyword lists, automated Scopus keyword lists, and abstracts. Text data were cleaned by removing punctuation, converting to lowercase, and identifying root words linking words with similar meaning (e.g., *biodiversity*, *biodiverse*). Words unhelpful in distinguishing topics were removed, including those lacking substantive meaning (e.g., *and*, *of*, copyright statements) or that were common across all topics (e.g., *study*, *abstract*). Next, the topics addressed by the publications were modelled using structural topic modelling [43, 44], with R packages stm, tm, quanteda, SnowballC and tidytext [45]. This approach supposes that every document addresses one or more topics, emphasising some more than others; each topic has characteristic word use compared to others. The topic areas are not defined by the analyst, but emerge organically from the language in the documents.

These models require the user to specify the number of topics to be delineated. Possible solutions between K = 5–30 topics were canvassed. We chose a number of topics that best satisfied the trade-off between semantic coherence (the tendency for topic words to co-occur in documents) and exclusivity (the tendency for topics to be characterised by words not used in other topics). Another consideration was the predictive value of topics for determining the likelihood of encountering, within a document, a word withheld from the formulation of the model (the heldout likelihood).

The prevalence of different topics was modelled as a function of year group, treating the groups as unordered factors. Where a topic matched an *a priori* expectation of an increase or decrease in prevalence, we tested each five-year interval against baseline (1981–1985), correcting for multiple comparisons [46]. For any remaining topics for which we had no *a priori* expectation, an exploratory test was conducted to search for differences between intervals, after correcting for multiple comparisons.

### Use of ANHM research outputs

Data on the national and institutional affiliations of authors citing documents published by ANHM affiliated authors between 1981–2020 were obtained from Scopus. Once the documents published by ANHM authors in each year group were identified, all records in a year group were selected and the ‘cited by’ option was chosen to display all documents that had cited any document authored by an ANHM affiliate during the year group specified. The authors of these documents were designated citing authors.

Once the citing documents were identified for a year group, the option ‘Documents by country/territory’ was selected to display the national affiliations of citing authors. The data are displayed as the number of documents with at least one author from each national affiliation, not as absolute counts of all authors. For example, if a document had three USA authors, two Spanish authors and an Australian author, it would be noted as a document with authors from those three countries, but the number of authors from each country would not be counted. The list of citing countries truncates at n = 160, so we used tighter searches to keep the list shorter than that, so as not to miss any citations. For example, when we searched for all the documents published in the year group 2016–2020 the list of citing countries exceeded 160, so we downloaded separate lists of nations for citing documents published in each year between 2016 and 2020, which generated less than 160 nations in each list. We then merged the results into lists of citation counts by national affiliation for the year group 2016–2020. For each year group, we present the total countries represented in the citing authors, the total citing documents, the mean number of citations/published ANHM document and, for Australia, the percentage of citing documents including at least one Australian author.

For each of the eight year groups, the countries of citing authors were ranked in order from the largest to the smallest. The top 20 countries in each year group were identified and a list compiled of the 27 countries ranked in the top 20 of any year group. Spearman rank correlation coefficients were used to test the hypothesis that the countries were ranked in the same order in each year group. This produced a matrix of 28 rank correlation coefficients, with the order in each year group compared against all other year groups. Therefore, a sequential Bonferroni correction was used to correct the *p*-values to ensure that the error rate across all 28 correlations was set at 0.05 [46].

Affiliations for citing authors were obtained using the option ‘Documents by affiliation.’ The number of research organisations in the world is much larger than the number of countries, so it was not possible to get untruncated lists without doing a very large number of (narrower) searches. Instead, we noted the top 50 institutions, based on the number of citing documents that had at least one author from that institution, for each year group and then grouped these institutions into the categories (i) an Australian museum, (ii) an Australian university, (iii) any other Australian address (e.g., government departments, private research institutes or businesses, private individuals), (iv) an international museum, (v) an international university (including museums coming under the umbrella of a university), and (vi) any other international address (e.g., government departments, private research institutes or businesses, private individuals).

## Results

### Research outputs of the ANHMs

**Fig 1:**
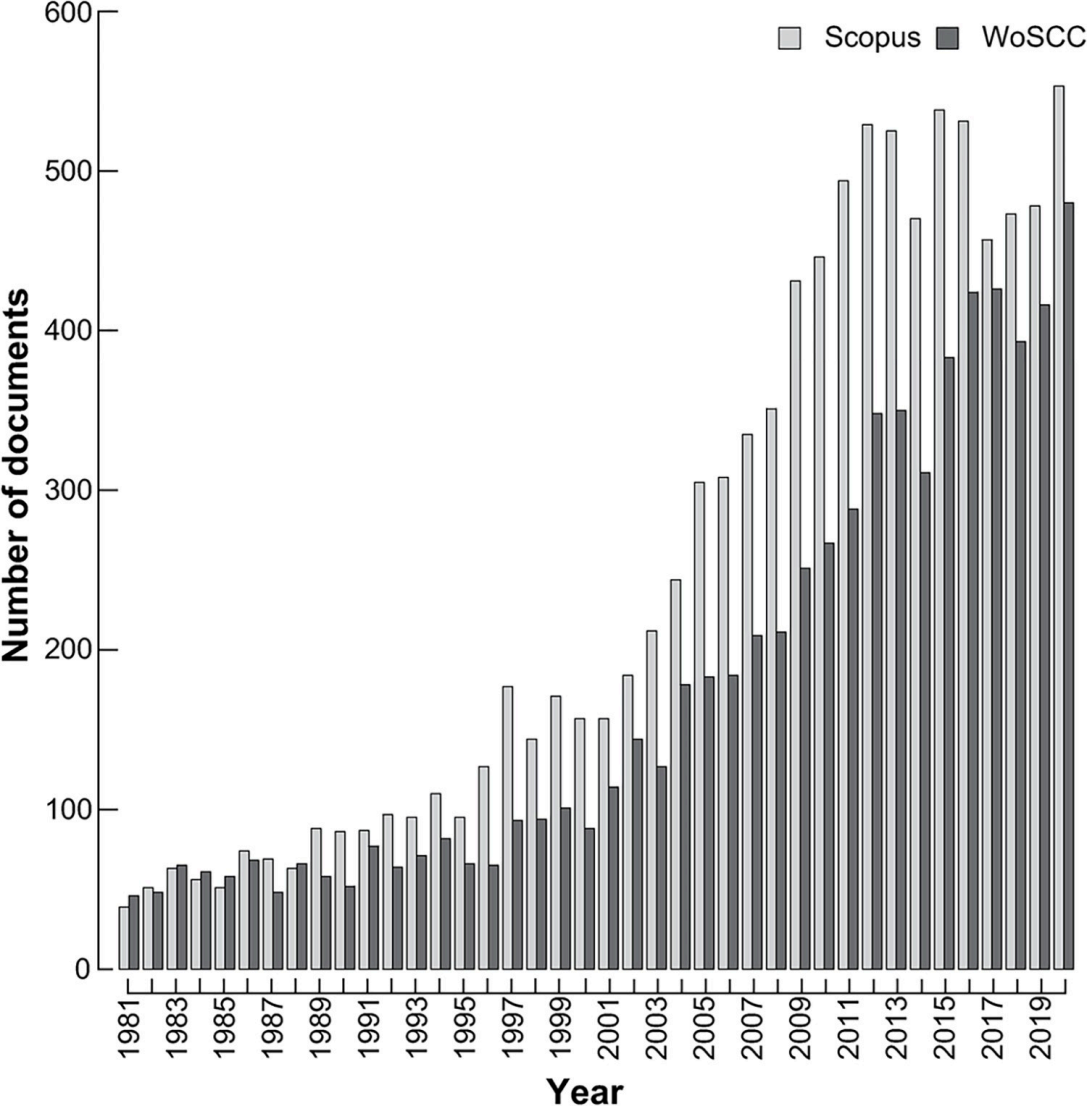
The number of published documents including at least one author with an ASNHM address 1981–2020, as identified by Scopus and Web of Science Core Collection.

**Fig 2:**
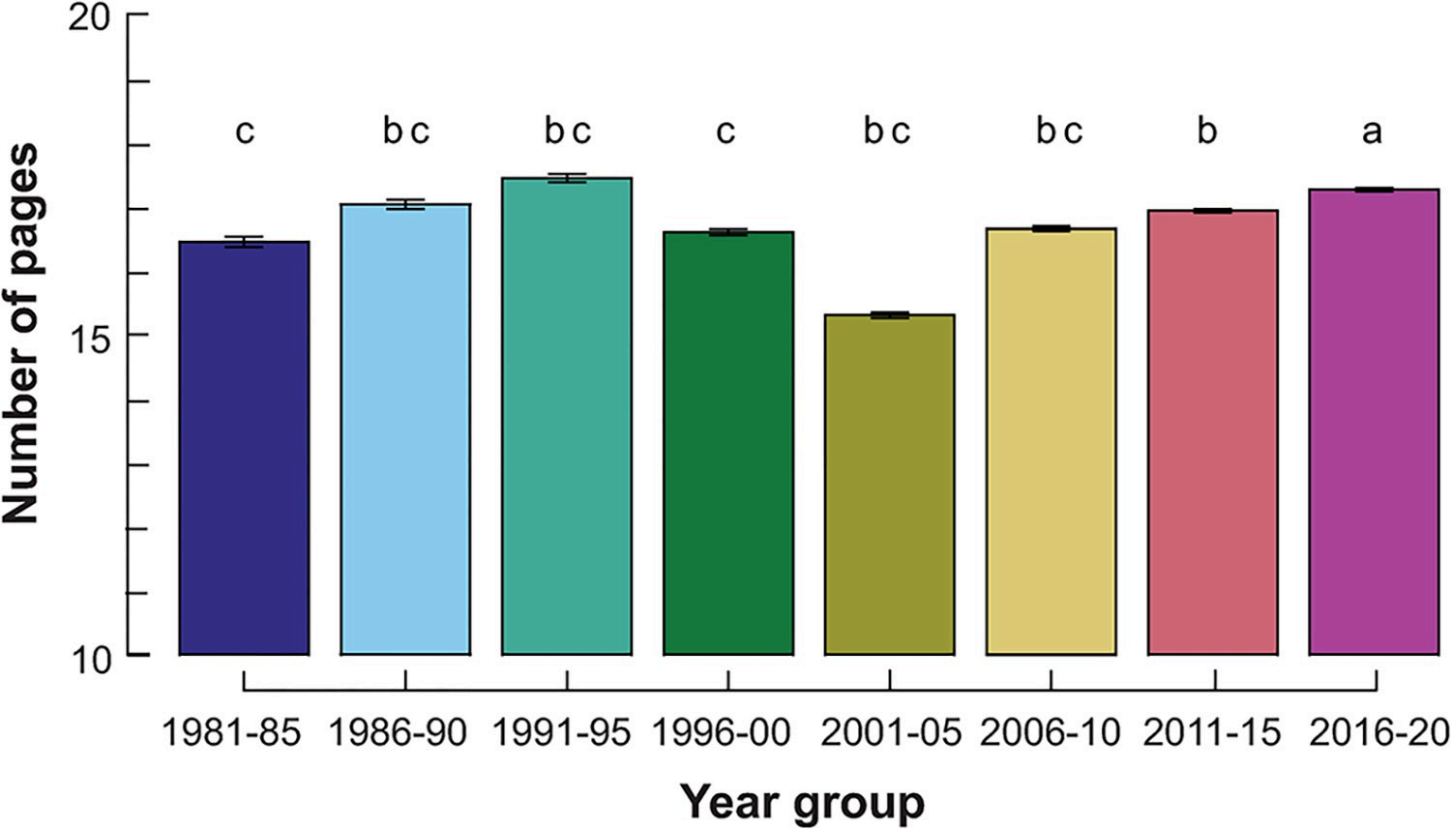
The mean document length in pages ± standard errors for ANHM publications 1981–2020, as identified by Scopus. Lettering indicates significantly different groupings at *p* < 0.05 as shown by Tukey HSD tests.

After removal of duplicates, Scopus retrieved 9,923 documents with at least one author claiming an ANHM affiliation 1981–2020. There was a strong upward trend in the number of documents from 40 in 1981 to a peak of 545 in 2020. When the data were split into four decades (1981–1990 etc.) and each decade analysed for a trend using the Mann-Kendall non-parametric test for trends, the first three decades all showed a significant increase (*p* < 0.005), while there was no trend from 2011–2020 (S = 3, *p* = 0.431). Qualitatively, the results of a WoSCC search gave similar results, albeit with lower numbers given the selectivity of WoSCC (Fig 1). Therefore, the pattern of rising numbers of documents with time was not an artefact of incomplete coverage by Scopus in earlier years. We obtained page lengths for 9,693 of 9,923 documents. The length of published documents differed significantly across the year groups (F_(7, 9685)_ = 10.12, *p* < 0.0001), although the range from a low of 15.3 in 2001–2005 to a high of 17.4 in 1991–1996 was small (Fig 2).

Most documents published in each year group were articles, which comprised 82.9% or more of the documents in any year group. The percentage of articles did decline over time, from 98.1% of documents in 1981–1985 to 82.9% in 2016–2020. Reviews, the next largest category, increased from 0.4% of documents in 1981–1985 to 8.3% in 2016–2020. Books were not a large component of the output, appearing in only the last four year groups and contributing less than 1% of output in each. Book chapters were also infrequent, again appearing in only the last four year groups and contributing between 1% and 3% of output (Table 1).

**Table 1:** The Scopus classification of documents published by the ANHMs 1981–2020.

Document type	1981–1985		1986–1990		1991–1995		1996–2000		2001–2005		2006–2010		2011–2015		2016–2020		Total	
	**N**	**%**	**N**	**%**	**N**	**%**	**N**	**%**	**N**	**%**	**N**	**%**	**N**	**%**	**N**		**N**	**%**
**Article**	255	98.1	366	96.3	470	97.1	719	92.7	937	85.0	1613	86.2	2144	83.9	2067	82.9	8571	86.4
**Review**	1	0.4	5	1.3	3	0.6	20	2.6	74	6.7	122	6.5	161	6.3	208	8.3	594	6.0
**Book chapter**	0	0.0	0	0.0	0	0.0	0	0.0	12	1.1	39	2.1	75	2.9	60	2.4	186	1.9
**Conference paper**	0	0.0	3	0.8	1	0.2	13	1.7	41	3.7	40	2.1	56	2.2	36	1.4	190	1.9
**Note**	3	1.2	3	0.8	3	0.6	10	1.3	12	1.1	15	0.8	31	1.2	34	1.4	111	1.1
**Letter**	1	0.4	3	0.8	5	1.0	3	0.4	3	0.3	17	0.9	52	2.0	48	1.9	132	1.3
**Short survey**	0	0.0	0	0.0	1	0.2	8	1.0	7	0.6	10	0.5	5	0.2	9	0.4	40	0.4
**Editorial**	0	0.0	0	0.0	1	0.2	3	0.4	11	1.0	3	0.2	20	0.8	14	0.6	52	0.5
**Erratum**	0	0.0	0	0.0	0	0.0	0	0.0	4	0.4	7	0.4	7	0.3	12	0.5	30	0.3
**Book**	0	0.0	0	0.0	0	0.0	0	0.0	1	0.1	5	0.3	5	0.2	4	0.2	15	0.2
**Total**	260	100	380	100	484	100	776	100	1102	100	1871	100	2558	100	2492	100	9923	100

The percentage of Open Access documents varied between 5.8% and 9.2% between 1981 and 2000, before rising steadily afterwards from 16.9% of documents in 2001–2005 to 42.9% of documents in 2016–2020. Similar growth in OA occurred for Australian research documents as a whole. Consistently, the largest category was green OA, in which authors archive the accepted text version of their documents in an online repository or website and no fee is payable (Table 2).

**Table 2:** The frequency of open access documents published by the ANHMs 1981–2020, according to Scopus classifications. A document may have more than one OA status (e.g., it may be both Gold and Green if the author has both paid for Gold OA via the publisher and released the document via a repository). Where available from SCIMAGO (https://www.scimagojr.com/countrysearch.php?country=AU), data for Australia are included for comparison.

	1981–1985	1986–1990	1991–1995	1996–2000	2001–2005	2006–2010	2011–2015	2016–2020	Total
**All Open Access**	24	22	35	69	186	476	792	1070	2674
**Gold** ^**1**^	2	0	3	1	4	44	215	459	728
**Hybrid Gold** ^**2**^	0	0	0	1	4	5	25	60	95
**Bronze** ^**3**^	11	16	14	49	104	225	328	340	1085
**Green** ^**4**^	13	10	19	32	109	354	591	797	1923
**N documents in that year group**	260	380	484	776	1102	1871	2558	2492	9923
**Percentage of all documents OA**	9.2	5.8	7.2	8.9	16.9	25.4	31.0	42.9	26.9
**Percentage of all documents OA for Australia**	-	-	-	18.6	23.6	28.6	36.4	47.6	-

^1^Gold (published by a dedicated OA publisher).

^2^Hybrid Gold (published in a subscription journal where authors may choose to publish OA).

^3^Bronze (the publisher has chosen to provide temporary or permanent free access).

^4^Green (the author’s version of an accepted document deposited in an online repository).

The mean number of authors per document increased across the year groups from 1.80 ± 0.07 in 1981–1985 to 6.09 ± 0.32 in 2016–2020 (mean ± SE), while the median number of authors also increased from 1.0 ± 1.0 in 1981–1985 to 3.0 ± 3.0 in 2016–2020 (median ± IQR). The number of unique authors rose across the year groups from 248 in 1981–1985 to 8,369 in 2016–2020 (Table 3). The discrepancy between means and medians indicates that the means are influenced by outlier documents with many authors. Eleven documents had over 100 authors each, one in 2006–2010, four in 2011–2015 and six in 2016–2020. ANOVA, with p set at 0.01 because of unequal variances, confirmed that the means of the year groups differed (F_(7, 9915)_ = 30.35, *p* < 0.0001). The percentage of documents with an ANHM first author declined from a high of 76.2% in 1981–1985 to 29.3% in 2016–2020 (Table 4).

**Table 3:** The mean and median number of authors and the number of unique individual authors of documents published by the ANHMs 1981–2020, based on Scopus data.

	1981–1985	1986–1990	1991–1995	1996–2000	2001–2005	2006–2010	2011–2015	2016–2020	All Years
**Mean**	1.80	2.25	2.29	2.50	2.85	3.81	4.44	6.08	4.15
**Standard error**	0.07	0.09	0.08	0.06	0.06	0.13	0.15	0.32	0.09
**Median**	1.0	2.0	2.0	2.0	2.0	3.0	3.0	4.0	3.0
**Interquartile range**	1.0	2.0	2.0	2.0	2.0	2.0	3.0	3.0	3.0
**N of unique authors**	248	468	580	994	1582	3537	5548	8369	16011
**N documents in that year group**	260	380	484	776	1102	1871	2558	2492	9923

**Table 4:** The percentage of documents in each year group where the first author is from an ANHM.

	1981–1985	1986–1990	1991–1995	1996–2000	2001–2005	2006–2010	2011–2015	2016–2020	All Years
**Number of documents with ANHM 1**^**st**^ **author**	198	282	336	481	567	776	936	731	4307
**Number of documents without ANHM 1**^**st**^ **author**	62	98	148	295	535	1095	1622	1761	5616
**% documents with ANHM 1**^**st**^ **author**	76.2	74.2	69.4	62.0	51.5	41.5	36.6	29.3	43.4
**Total number of documents**	260	380	484	776	1102	1871	2558	2492	9923

#### Collaboration analysis: Who co-publishes with ANHM researchers?

**Fig 3:**
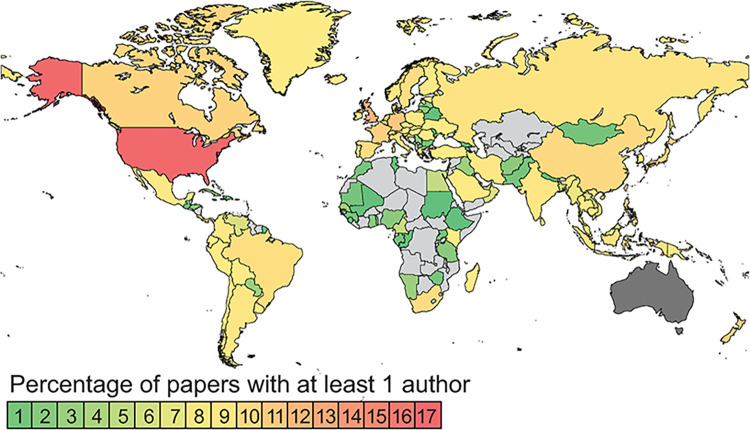
The percentage of documents published by ANHM authors 1981–2020 that included at least one co-author from a country other than Australia. ANHMs are based in Australia, so there is at least one Australian author on all these documents. Therefore, Australia is shown in dark grey to indicate that Australian authors are excluded. The map was produced using MapChart software’s free version licence https://www.mapchart.net/terms.html#licensing-maps, under a CC BY license with permission of Minas Giannekas, founder and developer of MapChart.

The percentages of documents in the different categories of collaboration all increased substantially with time. The lowest proportional increase was in collaborations with other Australian institutions (i.e., not other ANHMs or Australian universities), which nevertheless more than doubled between 1981–1985 and 2016–2020. The greatest proportional increase was in collaboration with international institutions other than museums or universities, which increased nearly tenfold. In 2016–2020, 87.2% of documents involved collaboration of some form. The pattern of increasing international collaboration was very similar to that for Australian publications as a whole (Table 5). The USA and the UK were included in the top five nations for international co-authors in all year groups, with a range of many other nations across the world occupying other positions (Table 5, Fig 3). Examining the data by year groups reveals a broadening of the range of collaborating nations over time (S2 Fig).

**Table 5:** The number and percentage of documents published by ANHMs including co-authors from other institutions, 1981–2020, based on Scopus data. One document might have co-authors from multiple categories, so the sum of documents across collaborations may exceed all documents for a year group. Percentages for each collaboration type are calculated as the percentage of the number of documents in each year group. Where available from SCIMAGO (https://www.scimagojr.com/countrysearch.php?country=AU), data for Australia as a whole are included for comparison.

Co–author from	1981–1985		1986–1990		1991–1995		1996–2000		2001–2005		2006–2010		2011–2015		2016–2020		Total	
	N	%	N	%	N	%	N	%	N	%	N	%	N	%	N	%	N	%
**Other Australian museum**	4	1.5	5	1.3	10	2.1	10	1.3	29	2.6	78	4.2	95	3.7	136	5.5	367	3.7
**Australian university**	37	14.2	72	18.9	112	23.1	215	27.7	405	36.8	874	46.7	1294	50.6	1534	61.6	4543	45.8
**Other Australian collaboration**	24	9.2	53	13.9	60	12.4	115	14.8	164	14.9	334	17.9	525	20.5	575	23.1	1850	18.6
**International museum**	8	3.1	17	4.5	29	6.0	65	8.4	74	6.7	209	11.2	344	13.5	410	16.5	1156	11.6
**International university**	13	5.0	43	11.3	35	7.2	136	17.5	274	24.9	595	31.8	997	39.0	1131	45.4	3224	32.5
**Other international collaboration**	7	2.7	17	4.5	30	6.2	75	9.7	128	11.6	341	18.2	518	20.3	652	26.2	1768	17.8
**Any collaboration**	74	28.5	157	41.3	225	46.5	445	57.3	714	64.8	1424	76.1	2036	79.7	2172	87.2	7247	73.0
**Any international collaboration**	26	10.0	66	17.4	92	19.0	222	28.6	381	34.6	837	44.7	1256	49.1	1431	57.4	4311	43.4
**% international collaboration for Australia**		-		-		-		28.7		33.7		39.4		44.9		54.7		-
**Top 5 countries for international co-authors**	USA, UK, New Zealand, France, Canada/China (tied)		USA, UK, New Zealand, Netherlands, Canada/China (tied)		UK, USA, New Zealand, Canada, Germany, France/New Zealand (tied)		USA, UK, New Zealand, Canada, Germany		USA, UK, Germany, New Zealand, France		USA, UK, Germany, Japan, New Zealand		USA, UK, Germany, France, New Zealand		USA, UK, Germany, France, New Zealand			
**N of documents in the year group**	260		380		484		776		1102		1871		2558		2492		9923	

#### Subject areas covered

**Fig 4:**
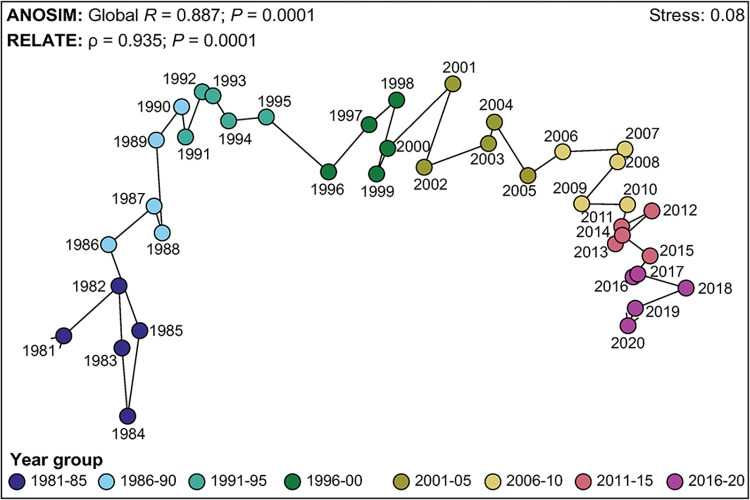
nMDS plot constructed from the square-root transformed percentage contribution of documents to sources in each of forty years between 1981 and 2020. Years are allocated to eight year groups each comprising five years. Line denotes the sequential trend over time.

*Source analysis*. Museum staff published in different sources over time (ANOSIM *p* = 0.001; Global *R* = 0.887). Each of the 28 pairwise comparisons differed significantly, with the biggest differences between year groups prior to 1996 vs those after 2006 (*R*-statistics = 1.000) and the smallest those between sequential year groups (*R*-statistics typically ≤ 0.680; S5 Table). This is illustrated on the associated nMDS plot, where the years representing the 1980s are located on the left-hand side of the plot and follow a curvilinear trend through time to those on the right-hand side (2010s) (Fig 4). The RELATE test indicated marked seriation over time (*p* = 0.001; rho = 0.935).

**Fig 5:**
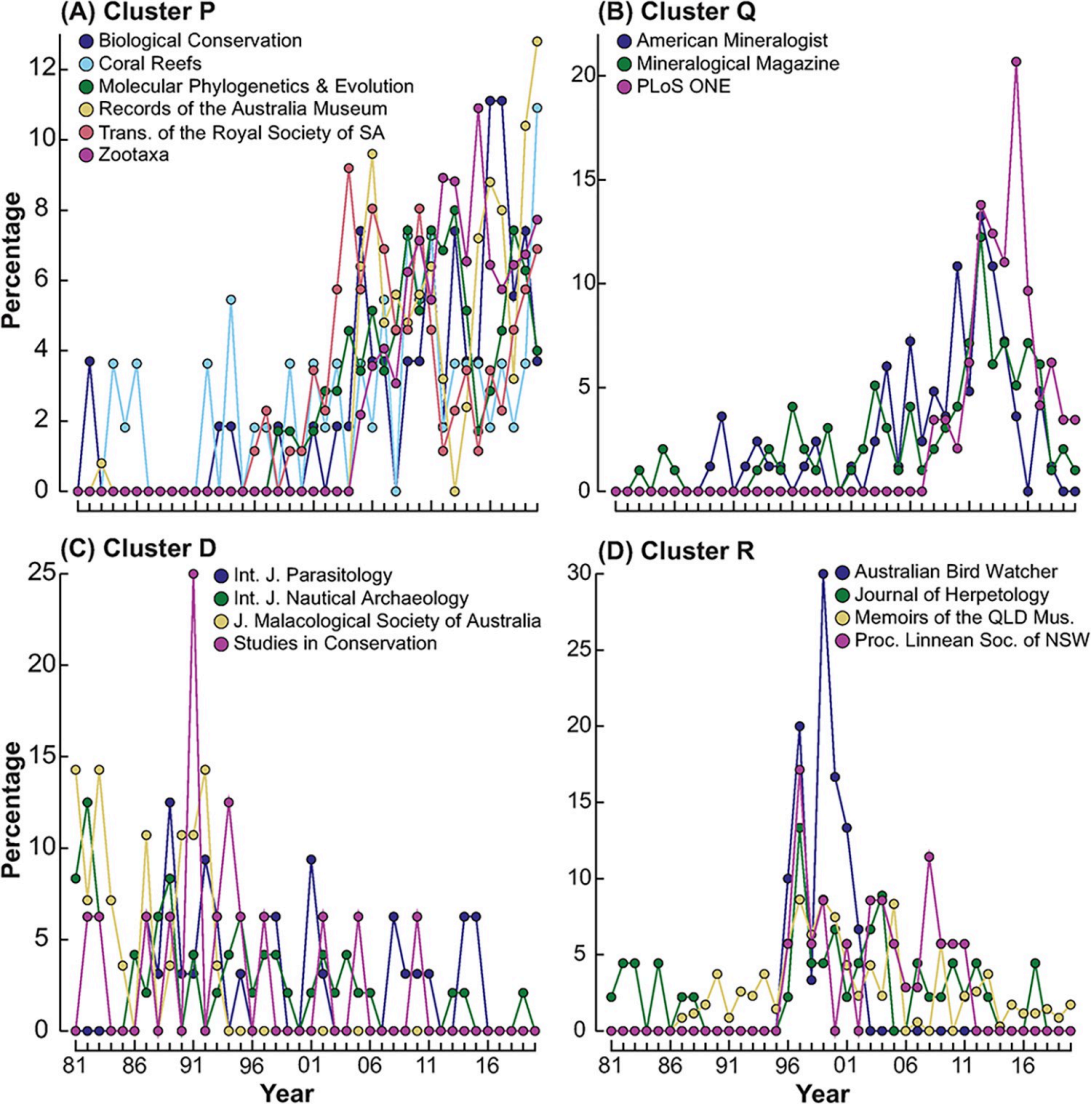
Line plots of four of the coherent groups of sources (a) P, (b) Q, (c) D and (d) R, identified by Type 3 SIMPROF tests. Each plot shows, for each source, the percentage contributions of documents to the total across the forty years.

The ‘coherent species curves’ routine indicated which journals were driving the changes. It identified 19 clusters of sources whose patterns in the relative number of documents published per year were similar over the forty years between 1981 and 2020 and significantly different (*p* < 0.05) from those sources in all other clusters (S1 Fig). Of these 19 clusters, nine contained a single source and thus were considered outliers. Of the ten remaining clusters, annual trends in publications are shown for four, i.e. Clusters D, P, Q and R (Fig 5). The six sources in Cluster P were those where documents were scarcely published prior to 1996, but those contributions of publications increased essentially sequentially annually. This group included *Zootaxa*, *Biological Conservation* and *Molecular Phylogenetics & Evolution*. The relative contribution of documents published in sources in Cluster Q also increased over time, however, this upward trend declined in the most recent years. This is illustrated by *PLoS One*, with the first museum authors publishing in this journal in 2008; those documents reaching a peak in 2015 (21% of all ANHM *PLoS One* documents) before declining to ~3% in 2019 and 2020. The relative number of documents published in sources in clusters D and R both declined over time. However, publications from sources in Cluster D, e.g., the *International Journal of Nautical Archaeology*, peaked in the 1980s, whereas those in Cluster R, e.g., *Australian Bird Watcher*, were low in the 1980s, peaked in the 1990s and subsequently declined (Fig 5). These results provide qualitative support for the predictions that the disciplines ‘genetics and molecular biology’ and ‘biological conservation’ would increase over time, given that the journals *Molecular Phylogenetics and Evolution* and *Biological Conservation* were included in Cluster P where new journals became prominent over the period of the study. The inclusion of *Studies in Conservation* in Cluster D (declining) does not contradict this conclusion, because this journal covers conservation of artefacts and not conservation biology. No computer science journals were included, so there was no evidence for the prediction that publications in computer science would increase. The evidence is ambivalent for the predictions that traditional core areas of ANHM work such as taxonomic descriptions and rocks and minerals would decline. The taxonomic journal *Zootaxa* and the *Records of the Australian Museum* were included in Cluster P where journals were increasing their share of publications, while *Memoirs of the Queensland Museum* was in Cluster R (declining) and *American Mineralogist* and *Mineralogical Magazine* were in Cluster Q (declining).

**Fig 6:**
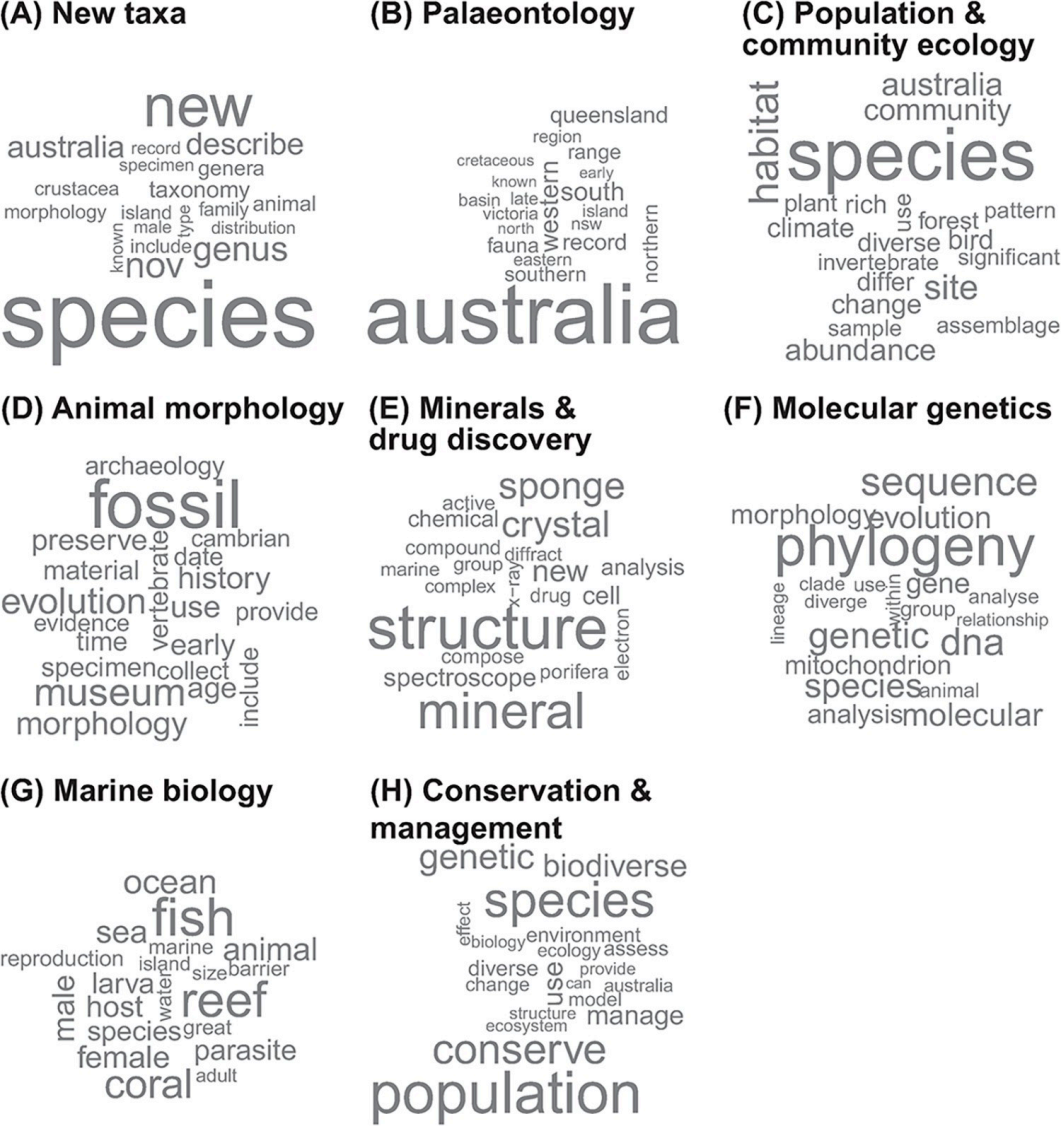
Word clouds illustrating words prominent in the identification of eight prominent topics in ANHM research. The size of words is relative to prevalence within clouds but not between them.

**Fig 7:**
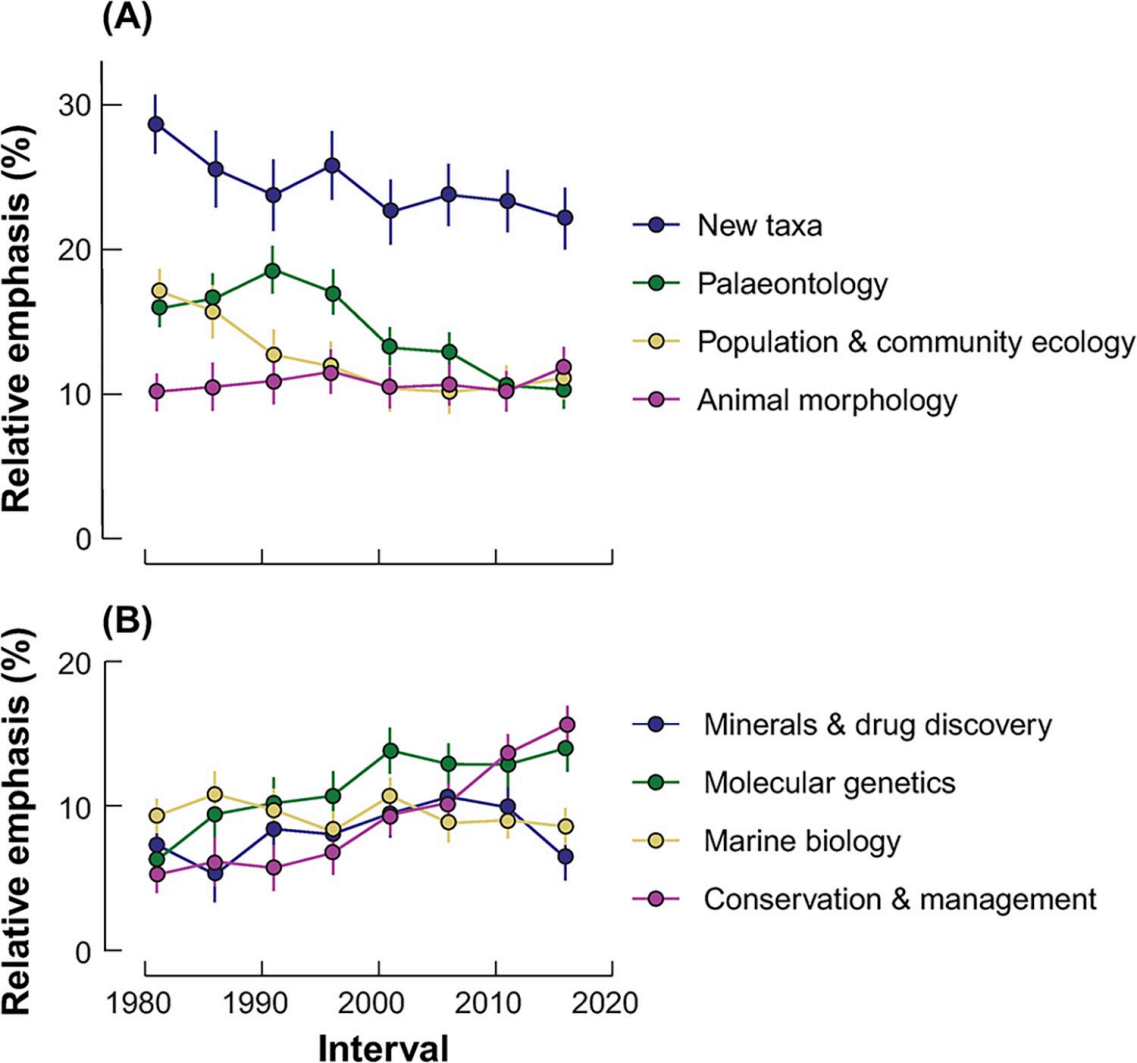
Changes in the relative frequency of eight topics in ANHM research over time. Error bars show standard errors.

*Structural topic modelling*. After text mining the words contained in document titles, keyword lists and abstracts, we settled on eight underlying topics that best balanced semantic coherence (the tendency for words within a topic to co-occur) and exclusivity (the tendency for a topic to contain words not found in other topics). Examining the words and documents most closely associated with each topic, we named these subject areas: New Taxa, Palaeontology, Population/Community Ecology, Animal Morphology, Minerals and Drug Discovery, Molecular Genetics, Marine Biology, and Conservation/Management (Fig 6). Changes in the relative frequency of the eight topics over time (Fig 7) found that two increased as predicted (Molecular Genetics, Conservation/Management) and two decreased as predicted (New Taxa, Palaeontology), although New Taxa remained the largest topic throughout the 40 years. Animal Morphology and Minerals and Drug Discovery remained stable and did not decrease as predicted, Population/Community Ecology decreased rather than increasing as predicted, and Marine Biology, for which no explicit prediction was made, remained stable (Table 6).

**Table 6:** *P*-values from bootstrapped tests for differences in topic prevalence relative to baseline (1981–1985), corrected for each topic using sequential Bonferroni.

Topic #	1	2	3	4	5	6	7	8
Description	New taxa	Palaeontology	Population / Community Ecology	Animal morphology	Minerals and drug discovery	Molecular genetics	Marine biology	Conservation /management
**Prediction**	decrease	decrease	increase	decrease	decrease	increase	–	increase
**Observed**	decrease	decrease	decrease	stable	stable	increase	stable	increase
**1986–1990**	0.333	1.000	0.411	1.000	1.000	0.062	1.000	1.000
**1991–1995**	0.133	0.411	0.012	1.000	1.000	0.054	1.000	1.000
**1996–2000**	0.333	1.000	***0*.*005***	1.000	1.000	**0.017**	1.000	1.000
**2001–2005**	**0.034**	0.122	***<0*.*001***	1.000	0.979	***<0*.*001***	1.000	**0.015**
**2006–2010**	0.078	0.067	***<0*.*001***	1.000	0.324	***<0*.*001***	1.000	***0*.*001***
**2011–2015**	0.058	***<0*.*001***	***<0*.*001***	1.000	0.615	***<0*.*001***	1.000	***<0*.*001***
**2016–2020**	**0.015**	***<0*.*001***	***<0*.*001***	0.722	1.000	***<0*.*001***	1.000	***<0*.*001***

Plain text *p* > 0.05, bold *p* < 0.05, bold and italic *p* <0.01

Overall, there have been shifts in the subjects covered in ANHM publications over time. While source analysis and topic modelling both indicate the emergence of molecular biology and biological conservation as growing areas of research, descriptions of extant and extinct taxa, as well as mineral research, remain large contributors.

### Use of ANHM research outputs

Across the year groups, authors from between 124 and 211 countries cited documents published by ANHM affiliated authors (note that ‘country’ in the database will include all examples of name changes over the 40 years of the study, as well as sometimes constituents of larger units such as the countries of the UK, so the upper limit in our data exceeds the number of sovereign states currently recognised in the world). The mean number of citations/document ranged from 11.1 ± 0.82 in 2016–2020 to 34.8 ± 2.08 in 2001–2005 (mean ± SE). The percentage of citing documents including at least one Australian author ranged from 25.5% in 1996–2000 to 36.0% in 1986–1990 (Table 7).

**Table 7:** The most common national affiliations of authors citing documents published by ANHM authors, 1981–2020, based on Scopus data. The figures are the number of citing documents with at least one author from that country in the author list. The list of 27 countries was compiled by including every country that occurred in the highest ranked 20 countries in any year group. For Australia, the percentage of citing documents with at least one Australian author is shown in parentheses.

Country	1981–1985	1986–1990	1991–1995	1996–2000	2001–2005	2006–2010	2011–2015	2016–2020
**Australia**	2035 (36.0%)	2877 (39.0%)	3353 (35.1%)	4274 (25.5%)	8308 (27.8%)	11251 (25.8%)	12721 (30.0%)	5309 (27.9%)
**United States**	1456	1711	2301	3606	8477	12156	12055	4757
**United Kingdom**	593	706	1097	1280	3161	4854	5176	2149
**Canada**	324	292	459	660	1724	2384	2431	1007
**Germany**	296	415	652	914	2481	3638	4111	1591
**France**	252	436	500	693	1740	3088	3278	1201
**New Zealand**	205	267	345	463	1151	1503	1647	585
**China**	196	239	366	477	1753	3189	3821	1388
**Spain**	187	215	346	455	1353	2474	2380	914
**Brazil**	155	239	260	356	1332	2882	2371	901
**Japan**	153	222	307	445	1199	1647	1854	671
**Italy**	119	152	254	281	1057	1678	1888	705
**Argentina**	106	126	192	259	669	1018	797	277
**Sweden**	105	138	201	269	743	984	1127	478
**South Africa**	100	115	179	243	671	1190	1332	473
**Russian Federation**	82	232	221	199	586	988	1338	447
**India**	75	128	174	170	605	881	858	362
**Israel**	70	80	43	72	211	330	338	172
**Switzerland**	69	75	155	147	607	1076	1031	454
**Netherlands**	67	102	146	248	498	902	1099	444
**Belgium**	61	76	87	145	429	799	927	398
**Norway**	57	48	100	106	352	623	814	346
**Mexico**	52	85	132	147	520	968	821	334
**Poland**	51	117	137	127	408	498	716	284
**Denmark**	50	86	100	132	437	722	753	312
**Czech Republic**	39	67	100	118	389	630	825	229
**Portugal**	29	53	91	130	560	941	918	383
**Total countries in the year group**	124	129	144	156	195	211	209	200
**Total citing documents**	5651	7378	9558	16730	29873	43677	42438	19014
**N ANHM documents in that year group**	260	380	484	776	1102	1871	2558	2492
**Mean cites/ANHM document ± standard error**	24.7 ± 5.21	22.6 ± 1.63	23.7 ± 1.67	27.0 ± 1.84	34.8 ± 2.08	33.5 ± 2.08	21.9 ± 1.06	11.1 ± 0.82

**Fig 8:**
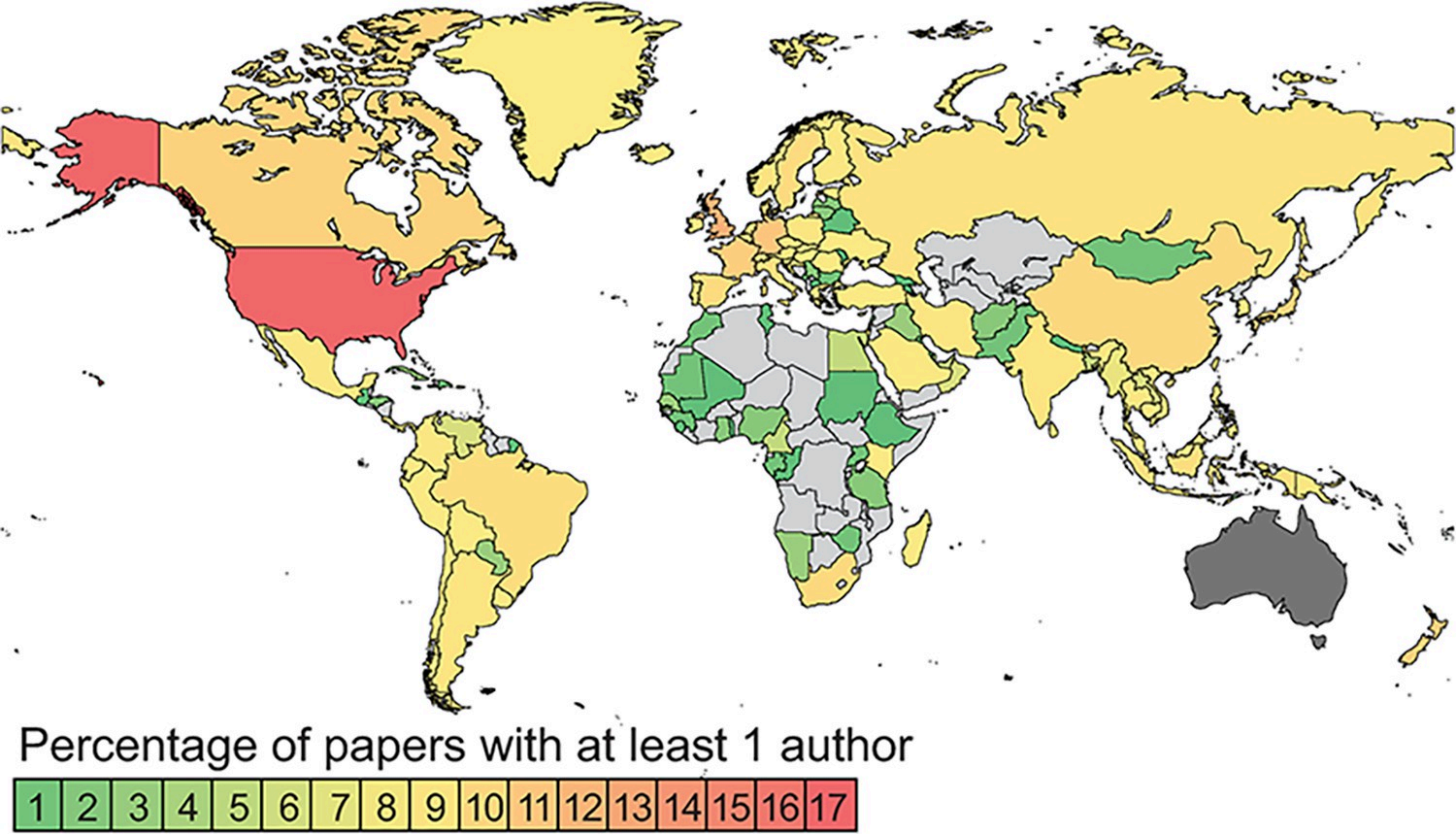
The percentage of documents citing work published by ANHM authors 1981–2020 that included at least one author from the country indicated. The map was produced using MapChart software’s free version licence https://www.mapchart.net/terms.html#licensing-maps, under a CC BY license with permission of Minas Giannekas, founder and developer of MapChart.

Consistently across all the year groups, the top three rankings were Australia, USA and UK. They ranked in that order for all year groups except 2006–2010, when USA held first spot with Australia second (Table 7). Although there were fluctuations in places below the first three, they were minor and the rank correlations between all pairs of year groups were significant after sequential Bonferroni correction (Table 8). Considering the entire data set combined, the predominance of citations from Australia, USA and UK was strong (Fig 8). Examining the data by year groups reveals a broadening of the range of citing nations over time (S3 Fig).

**Table 8:** Spearman rank correlation coefficients for the order of countries in each year group, using data from Table 7.

Year group	1981–1985	1986–1990	1991–1995	1996–2000	2001–2005	2006–2010	2011–2015	2016–2020
**1981–1985**	1	0.930**	0.947**	0.946**	0.926**	0.900**	0.879**	0.881**
**1986–1990**		1	0.951**	0.933**	0.897**	0.857**	0.854**	0.845**
**1991–1995**			1	0.969**	0.960**	0.936**	0.916**	0.911**
**1996–2000**				1	0.970**	0.948**	0.928**	0.925**
**2001–2005**					1	0.979**	0.933**	0.941**
**2006–2010**						1	0.945**	0.941**
**2011–2015**							1	0.983**
**2016–2020**								1

** *p* < 0.001

Considering the top 50 institutional affiliations citing ANHM documents in each year group, Australian universities were consistently the largest affiliation, followed by international universities. All other categories of institutional affiliations were represented (Table 9).

**Table 9:** The number and percentage of the 50 highest ranked institutional affiliations of authors citing documents published by ASMNHs, 1981–2020, using Scopus data. Rankings are based on the number of citing documents that included at least one author from that affiliation.

Co-author from	1981–1985		1986–1990		1991–1995		1996–2000		2001–2005		2006–2010		2011–2015		2016–2020		Total	
	N	%	N	%	N	%	N	%	N	%	N	%	N	%	N	%	N	%
**Other Australian museum**	4	8	6	12	5	10	5	10	5	10	5	10	5	10	4	8	39	9.75
**Australian universities**	19	38	14	28	18	36	23	46	17	34	16	32	20	40	17	34	144	36
**Other Australian co-authors**	3	6	2	4	1	2	3	6	2	4	1	2	1	2	4	8	17	4.25
**International museum**	6	12	4	8	3	6	5	10	6	12	6	12	3	6	4	8	37	9.25
**International universities**	10	20	17	34	10	20	8	16	15	30	15	30	12	24	13	26	100	25
**Other international co-authors**	8	16	7	14	13	26	6	12	5	10	7	14	9	18	8	16	63	15.75
**N**	50	100	50	100	50	100	50	100	50	100	50	100	50	100	50	100	400	100

## Discussion

### Typologies of museum research

According to Sigfúsdóttir’s [5] typology of museum-based research, the peer-reviewed publications that form the basis of our evaluation are significant outputs in two categories: scholar-curator and laboratory. Both apply traditional research methods, often involve external collaborations, and have peer-reviewed publications as the most valued outputs. Of the other categories, empowering public knowledge or experience as an explicit component of knowledge generation (co-researching with communities) and the study of museums in their own right (museology) may generate traditional publications, but the imperative to do so is less. Practice-based research is linked to daily tasks, often having non-traditional outputs such as exhibitions. Thus, our analysis is an incomplete survey of ANHM research based on convenience, which should not be misinterpreted as valuing some categories of research over others.

### Output of the ANHMs

#### Increased productivity: Why and for how long?

The number of documents by authors claiming an association with one or more ANHMs grew over tenfold between 1981 and 2020. The increase was not because similar numbers of authors published more, because the number of unique authors rose at an even greater rate across the same period. Possible explanations include:

*Impacts of technology*. Changes in technology markedly influenced scientific publication over the study period, improving communications between authors and between authors and publishers through email [47] and interactive online technologies [48]. These technologies also facilitated writing and editing through multiple iterations of a manuscript [49, 50], improving access to data analysis [51, 52], and simplifying preparation of technical figures (as evidenced by the instructions for preparing figures in modern journals).

*Increased research collaborations*. There is likely increased use of adjunct or research associate positions by ANHMs, as well as collaborations involving research students at universities and researchers at other museums, government departments, or other organisations. The internet was recognised early for enabling collaboration [53] and, especially with the development of interactive Web 2.0 technologies, it has greatly increased opportunities [48]. One example is the comparative ease of drawing diverse authors together to produce special issues of journals, such as ‘Systematics and diversity of annelids’ edited jointly by Maria Capa, a Spanish academic, and Pat Hutchings, a researcher at the Australian Museum in Sydney [54]. Other authors highlight potential collaborations between museums and other public institutions in combining collections or archives [55–57].

*Increased staffing*. This is unlikely, given that there is an international trend for a reduction in curatorial staff employed at museums [58, 59], and for those who are engaged to have shorter employment terms, at least in Europe [60]. We lack the data to confirm any trend to smaller staffs who are mainly on short-term contracts in Australia, but Hutchings [61] noted that over the 10 years preceding her paper the research staff at the Australian Museum fell by 50% as staff retired or left when grant funding ended, returning staffing to 1960s levels.

*Changes in staff profile or expectations*. The profile of staff might be changing, though, with greater emphasis on research records when making appointments (especially number of publications and the impact factors of the journals), leading in turn to higher publication rates. Joint appointments between museums and universities, such as those reported by [62], might be important too. If these changes are occurring, they may represent a focus on ‘… research in the strictest of academic terms–typically collection-based research disseminated in peer-reviewed publications… (rather than) an open-ended, practice-based and experimental process, characterised by self-reflectivity and interaction with visitors’ [5], p. 1.

*Continued contributions from retired staff*. Retired staff, who frequently continue to work in an honorary capacity, contribute to museums’ research outputs [58].

*Volunteer contributions*. The annual reports of the ANHMs acknowledge the value of volunteers to their missions and achievements. For example, in its Strategic Plan 2016–2021 the Tasmanian Museum and Art Gallery logged 8,670 volunteer hours in 2019–2020 [63]. The Western Australian Museum refers to ‘348 dedicated volunteers’ [64] and the Australian Museum logged 30,500 hours by 276 volunteers in 2019–2020 [65]. This is similar to the strong volunteer culture reported at USA museums, but not in highly unionised European museums [59]. While not all volunteers will contribute directly to scholar-curator or laboratory-based research projects, their assistance frees time for staff to research or covers tasks once undertaken by staff who were not replaced.

We are unsure if growth can continue, with the data already showing no increase in the decade 2011–2020. As they age, retired staff will decline in productivity, while not everyone works in retirement. Facilities may also reach saturation for people who can work simultaneously on a range of projects, although the increased sharing of digital information may help to overcome the limitations of physical facilities [66]. Unless funding for research staff increases or productivity is enhanced with new technology, maintaining productivity is likely to be more and more reliant on collaborations and the work of volunteers, requiring time to nurture collaborations.

#### Open access, types of documents, numbers of authors and author order

The increased percentage of documents published in some form of OA over time reflects a growing interest since the 1990s in making the results of scientific research, much of which is funded directly or indirectly by taxpayers, free to read for anyone instead of being restricted behind a paywall (e.g., [67–70])–a goal aligned with the communication objectives in the Annual Reports of many ANHMs. The growth in OA publishing by ANHMs reflects the general growth in OA [71], although use of Gold OA may lag behind the general trend–in 2009, 6.8% of Scopus publications were Gold OA [71], whereas between 2006 and 2010 ANHM publications included only 2.2% Gold OA. The ANHMs also appear to be lagging slightly behind the increase in OA publications for Australia as a whole, possibly because of budgetary constraints in paying Article Processing Charges (APCs) for OA documents, or difficulty in negotiating Read and Publish deals. The online Directory of Open Access Journals (DOAJ), which maintains an index of OA journals, includes an option to search for OA journals without APCs. At least one ANHM journal, *Records of the Australian Museum*, is included. OA could be a part of co-research with communities by ensuring that the outputs of research are available to all and not locked behind a paywall.

While most documents are articles, the proportion of reviews is growing. It is likely that books are underrepresented in the types of documents published because of changes in Scopus’ document inclusion policy. At its inception in 2004, Scopus included books and book chapters only when the book was part of a named series. From 2013, Scopus began including a much wider range of books and book chapters [39], increasing the chance that they would be reported in our data.

The increases in the mean number of authors per document from 1.80 ± 0.07 in 1981–1985 to 6.09 ± 0.32 in 2016–2020 (mean ± SE) follows a trend reported in many scientific fields [72, 73], although only one ANHM document with 497 authors approached the figures of 500 or even 5000 authors per document reported by [74]. Increased authorships reflect, at least partly, multidisciplinary collaborations on complex problems [75], as well as technological aids [48]. First authorship may indicate the author making the leading contribution, although this convention does not hold across all disciplines and may change with time [76]. Therefore, one must be cautious in interpreting the decline in ANHM first authorship from 76.2% in 1981–1985 to 29.3% in 2016–2020. Our view is that it reflects the increasing collaboration shown by growing author numbers.

### Collaboration analysis: Who co-publishes with ANHM researchers?

Collaboration is a large component of the laboratory category of museum research [5]. It varies from informal discussion and advice to active participation in collecting, analysing or publishing data, with the informal aspects not captured in bibliometric data. Nevertheless, the bibliometric data are ‘invariant and verifiable’ [75], underpin meta-analyses and systemic reviews [77–79], and do show that collaboration in ANHM publication has increased markedly over the past 40 years, although much of it remains with the large English speaking scientific communities in the USA and the UK. This is one example of the increased scientific collaboration noted in the late 20^th^ Century [75] that accelerated in the 21^st^ Century [80, 81], with claims that it shows no sign of abating [82]. The reasons behind the growth in collaboration in general likely apply to the work of ANHMs, too: cost efficiencies in use of specialised facilities (collections are an important example), declining travel and communication costs, the need to bring diverse specialist expertise together on a specific problem, and incentives from politicians and funding bodies investing in research or innovation [75].

Advantages of increased collaboration include more efficient use of specialised facilities (including collections), combining results from fieldwork in different locales, increased citations for documents involving collaborators across institutional or national boundaries (although some of this might simply be the well-known correlation between the number of authors and citations [83]), increased novelty in collaborative documents, strengthening of research networks, and greater success in funding applications [75, 80–82]. Downsides include the possibility that research may gravitate to a consensus on identifying important topics, leaving less popular areas starved for attention [80].

The ANHMs are well-placed to accentuate benefits and minimise disadvantages. Their collections are significant but localised assets (especially given Australia’s high endemism), requiring visits from researchers needing to use them or development of other collaborative networks to share data. Advances in digitising collections may reduce or even remove these impediments, or at the least allow researchers to decide if it is worth travelling to view a specimen [15, 84, 85]. Specialist skills such as taxonomy are rarer because of perceived poor career prospects [86, 87], so projects needing taxonomic expertise require collaboration [88, 89]. Globally, museum directors recognise collaborative potential too [59].

### Subject areas covered

ANHM authors published in different sources over time. Source analysis and topic modelling both supported the prediction of an increase in publications in molecular biology and conservation biology. Specialist molecular journals of particular relevance to natural history only appeared during the period of this study, as did many important conservation journals. Despite the increased outputs in these new areas, traditional strengths of ANHM research involving taxonomy and phylogeny remained large components of published research, although relative outputs declined slightly over time. This is in spite of challenges in availability of funding and restrictions on who can apply.

Research policies at museums likely had significant influence on the temporal changes observed in the journals where research was published and the topics covered. In one well-documented example, Frank Talbot, director of the Australian Museum in Sydney, established an environmental research department in 1968 to assist government agencies in identifying priority areas for biological conservation. The new department focused on bird ecology, mainly in New South Wales, with changes in staffing shifting emphasis from birds of forests and heaths to those of environments modified by human activity. Research focus at the Australian Museum shifted back to emphasising collection-based research by the 1990s, before the environmental department, after several name changes, was disestablished in 2011 [90].

Although there is growing interest in digitising collections and developing virtual displays [91, 92], publications on how to digitise collections or the use of digitised collections were not identified as major research interests by either source analysis or topic modelling, although this might reflect authors simply not using relevant terms in titles, abstracts or keywords or overlooking non-traditional research outputs, especially where people other than research staff are involved in digitisation projects [5].

### Use of ANHM research outputs

In all year groups except 2006–2010, ANHM outputs were cited mainly by Australians. While this is unsurprising considering the regional fauna and issues studied by the ANHMs, many countries were represented in the citation lists, so many documents are of international relevance.

Bibliometric studies give context to these figures. Meffe [93] proposed the concept ‘internationally recognised regional journal’ for a regional journal publishing documents cited internationally. Similarly, Tijssen et al. [94] and Tijssen [95] divided South African journals into those used internationally and those rarely cited outside South Africa (see also [34, 96, 97] for examples of Australian journals and books). ANHM research is important for Australia, while having international relevance as revealed by international citations.

### Enduring relevance

Natural history museums face many challenges, with salient ones highlighted in conversations involving Volker Mosbrugger (Director General of the Senckenberg Society for Natural Research) and Kirk Johnson, (Sant Director of the Smithsonian’s National Museum of Natural History) documented by [59]:

- Variations between countries in the culture of volunteering
- Respect for the provenance of objects, in particular a wish to decolonise museum collections by repatriating items of cultural significance
- Variations between countries in the role of museums in political topics
- A critical need to assess scientific and social issues such as climate change, racism and globalisation.
- Involving visitors in action by volunteering or citizen science
- Leveraging technology to increase collaboration, while recognising the unique cultures and reflecting those difference in tailored exhibits.

The conversations highlight the central role of displays and education in the work of museums, not least because they attract visitors. While collecting, preserving, and researching are vital museum functions too [1], they are harder to commoditise [98].

In that context, the increases in publications by authors affiliated with ANHMs between 1981 and 2020 point to an enduring relevance of natural history museum collections, despite a changing research environment and on-going reflection on the role of museums in science and society [2, 3]. On the scientific side, one example of a new role for collections is the application of molecular techniques to museum specimens to resolve cryptic species, which are very similar morphologically, but have significant genetic differences [99]. Innovatively, museum specimens also contribute to our understanding of ecotoxicology [100] and pathogen surveillance [101]. Developments such as Computerised Tomography (CT) scans may provide digital traces of internal structure of specimens [84], facilitating imagery necessary to measure body parts accurately when collections are shared online [102, 103]. This can reduce costs and administrative impediments in loaning specimens or travel to inspect them.

Digitising collections is one area where research and display converge, with greater visibility for diverse categories of research [5]. Educational research is exploring the potential of digitised collections to enhance learning [91, 92, 104, 105], moving displays closer to the goal of immersive collaboration. It also places museum research in the unique position of linking science, in the form of the research, with mainstream culture, in the form of the display [4, 5], while working toward the goal of internationally catalogued and accessible digitised collections [106].

## Supporting information

S1 Table Historical backgrounds and statements of research commitment from the ANHMs. (DOCX)

S2 Table The Scopus affiliation IDs used to search for documents with at least one ANHM author. (DOCX)

S3 Table The cleaned data file of ANHM outputs. (XLSX)

S4 Table The 2020 journal percentile rankings for all journals where ANHM staff published. (DOCX)

S5 Table *R*-statistic values from a one-way ANOSIM test on the sources of documents published by authors from the Australian Museum in various year groups. All pairwise comparisons differ significantly (*p* = 0.032–0.008). Shading from red to green denotes increasing value for the *R*-statistic.
(DOCX)

S1 File The protocol for using the ‘Advanced document search’ feature in Scopus to retrieve ANHM publications. (DOCX)

S1 Fig Dendrogram derived from cluster analysis using the percentage contributions of the documents in sources annually over forty years. The clusters to the left of each black vertical line represent sources whose temporal pattern of publication were shown by SIMPROF not to be significantly different from each other (*p >* 0.05), but to be significantly different from those in all other groups of samples (*p* < 0.05). * denotes sources that are edited books rather than journals.
(TIF)

S2 Fig The percentage of documents published by ANHM authors in each of eight year groups 1981–2020 that included at least one co-author from a country other than Australia. ANHMs are based in Australia, so there is at least one Australian author on all these documents. Therefore, Australia is shown in dark grey to indicate that Australian authors are excluded. The map was produced using MapChart software’s free version licence https://www.mapchart.net/terms.html#licensing-maps, under a CC BY license with permission of Minas Giannekas, founder and developer of MapChart.
(TIF)

S3 Fig The percentage of documents citing work published by ANHM authors in each of eight year groups 1981–2020 that included at least one author from the country indicated. The map was produced using MapChart software’s free version licence https://www.mapchart.net/terms.html#licensing-maps, under a CC BY license with permission of Minas Giannekas, founder and developer of MapChart.
(TIF)
