# Supplementary material for: Research publications of Australia’s natural history museums, 1981–2020: Enduring relevance in a changing world
Source: PLoS One. 2023 Jun 23;18(6):e0287659. doi: 10.1371/journal.pone.0287659 (PMC10289469; doi:10.1371/journal.pone.0287659)
Supplement: S1 Table — (DOCX) [file pone.0287659.s001.docx]

**S1 Table. Historical backgrounds and statements of research commitment from the ANHMs.**

| Museum | Historical notes | Research commitment |
| --- | --- | --- |
| Australian Museum | Despite the name, this is a state museum. It is Australia’s first museum, established in 1829 as the Sydney Museum. It became the Australian Museum in 1836. Today, the Australian Museum has significant roles in scientific research, education and cultural outreach. | The latest Annual Report (Australian Museum 2020) notes:  ‘The AM ... plays a leading role in taxonomic and systematic research, and facilitates significant research on coral reef ecology at the AM Lizard Island Research Station. ...  The AM is a dynamic source of reliable scientific information and a touchstone for informed debate about some of the most pressing environmental and social challenges facing our region: the loss of biodiversity, a changing climate, and the search for cultural identity.’ |
| Museum and Art Gallery of the Northern Territory | Founded in 1965. | According to the 2019/2020 Annual Report (MAGNT 2021):  ‘Our role is to:   - collect and preserve employing world best practice; - research and interpret with rigour and imagination; - facilitate scientific, artistic and cultural activity; and - communicate the stories of who and where we are. ‘ |
| Museum Victoria | Founded in 1854. | According to the 2020-21 Annual Report (Museums Board of Victoria 2021), there are two strategic objectives relevant to research:  ‘Strategic objective 2: Museums Victoria has the primary material collection that inspires and allows excellent enquiry into our region’s big contemporary and historical questions.  Strategic objective 4: Museums Victoria is a centre for technological and scientific expertise and fosters innovation to build economic value.’ |
| National Museum of Australia | The museum was founded by Commonwealth legislation in 1980, although it did not occupy permanent, purpose-built premises until 2001. | According to the 2020-21 Annual Report (National Museum of Australia 2021):  ‘The Museum’s exhibitions, collections, programs and research focus on 3 interrelated subject areas:   - First Nations peoples’ history and culture - Australian history and society since European settlement - Australian environmental history, including the history of human interaction with the land.’ |
| Queensland Museum | Planning began in 1859 and first public display opened in 1862. There is a network of centres: Queensland Museum in Brisbane, Museum of Tropical Queensland, Workshops Rail Museum, Cobb+Co Museum, Research and Loans Centre. | According to the 2020-21 Annual Report (Queensland Museum Network 2021):  ‘Vision  To be a valued and trusted museum network that creates authentic and compelling experiences.  Purpose  Connect real objects and contemporary research with communities and tell stories that inspire, enrich and empower. ‘ |
| South Australian Museum | The founding legislation was passed in 1856 and the buildings opened in 1861. | According to the 2020-21 Annual Report (South Australian Museum 2021), one of the Functions and Objectives is to: ‘Integrate, connect and leverage our collections and research to generate new knowledge.’ There are extensive references to research grants received, research publications, research collaborations, and involvement of staff in supervision of research students. |
| Tasmanian Museum and Art Gallery, incorporating the Tasmanian Herbarium | Established in 1846 by the Royal Society of Tasmania. | References to research are extensive throughout the Annual Report (2019-2020) (Tasmanian Museum and Art Gallery 2021). Two examples are:  ‘Strategic outcome 2  Strong collections that tell Tasmania’s stories  In 2019-20, TMAG continued to build its diverse collections through strategic acquisition and, in the natural sciences, targeted field work. These collections underpin our understanding of Tasmania’s unique natural environment, culture and heritage, as well as serving as the sources of new knowledge through research and scholarship. Much effort was also directed towards improving electronic access to collection data through preparations for migration to a new collection management system, anticipated in the next year. ‘  And  ‘Zoological research focuses on documenting Tasmanian faunal diversity and on systematic research on that fauna. Three key areas are: surveying and documenting the fauna of poorly known parts of Tasmania; collaborating on collections-based research with external academics and students; and producing research publications. ‘ |
| Western Australian Museum | Founded in 1891. Currently has seven public locations, including a Collections and Research Centre. | The latest Annual Report (2019-2020) (Western Australian Museum 2020), includes the following performance measure against the Strategic Plan:  ‘We will expand our international reputation for collections, research, public engagement and creativity.’  A value of the museum is:  ‘We will inspire people to explore our world and will advance knowledge through discovery, research and life-long learning.’  Furthermore:  ‘The WA Museum and its associates are prolific contributors to the knowledge and understanding of Western Australia’s natural environment, for the benefit of a range of communities and industries, in Australia and world-wide. These contributions range across the fields of science and technology, research and development, conservation, planning, development and education.’ |
