# Supplementary material for: Research publications of Australia’s natural history museums, 1981–2020: Enduring relevance in a changing world
Source: PLoS One. 2023 Jun 23;18(6):e0287659. doi: 10.1371/journal.pone.0287659 (PMC10289469; doi:10.1371/journal.pone.0287659)
Supplement: S2 Table — (DOCX) [file pone.0287659.s002.docx]

**S2 Table. The Scopus affiliation IDs used to search for documents published by the ANHMs.**

| 60017681 | Australian Museum |
| --- | --- |
| 101069927 | Museum and Art Gallery of the Northern Territory |
| 100316217 | Museum and Art Gallery of the Northern Territory |
| 113446170 | Museum and Art Gallery of the Northern Territory |
| 109654660 | Museum and Art Gallery of the Northern Territory |
| 60033221 | Museum Victoria |
| 118822893 | Museums Victoria |
| 60103171 | National Museum of Australia |
| 60087943 | Northern Territory Museum of Arts and Sciences |
| 60015600 | Queensland Museum |
| 60004899 | Museum of Tropical Queensland in Townsville |
| 60024485 | South Australian Museum |
| 60011086 | Tasmanian Museum and Art Gallery |
| 60017526 | Western Australian Museum |
