# Supplementary material for: Research publications of Australia’s natural history museums, 1981–2020: Enduring relevance in a changing world
Source: PLoS One. 2023 Jun 23;18(6):e0287659. doi: 10.1371/journal.pone.0287659 (PMC10289469; doi:10.1371/journal.pone.0287659)
Supplement: S4 Table — (DOCX) [file pone.0287659.s004.docx]

S4 Table. All sources in which ANHM authors published 1981-2020, together with the highest percentile ranking for each source in 2020.

| **Publication Title** | **Highest percentile ranking in Scopus 2020** |
| --- | --- |
| 18th International Corrosion Congress 2011 | Not available |
| 1994 SEG Annual Meeting | Not available |
| 2009 3rd IEEE International Conference on Digital Ecosystems and Technologies, DEST '09 | Not available |
| 2016 International Conference on Digital Image Computing: Techniques and Applications, DICTA 2016 | Not available |
| 40th Annual Conference Australian Society of Sugar Cane Technologists, ASSCT 2018 | Not available |
| 41st Annual Conference - Australian Society of Sugar Cane Technologists 2019 | Not available |
| 6th International Conference on Information Technology and Applications, ICITA 2009 | Not available |
| A Companion to Global Environmental History | Not available |
| Acarologia | 57% |
| ACM International Conference Proceeding Series | Not available |
| Acquiring Cultures: Histories of World Art on Western Markets | Not available |
| ACS Earth and Space Chemistry | 68% |
| ACS Omega | 73% |
| Acta Arachnologica | 9% |
| Acta Carsologica | 38% |
| Acta Chiropterologica | 58% |
| Acta Crystallographica Section B: Structural Science | Not available |
| Acta Crystallographica Section B: Structural Science, Crystal Engineering and Materials | 75% |
| Acta Crystallographica Section C: Structural Chemistry | 40% |
| Acta Crystallographica Section E: Crystallographic Communications | 36% |
| Acta Geologica Polonica | 46% |
| Acta Geologica Sinica | 48% |
| Acta Horticulturae | 15% |
| Acta Ichthyologica et Piscatoria | 43% |
| Acta Oceanologica Sinica | 44% |
| Acta Palaeobotanica | 51% |
| Acta Palaeontologica Polonica | 77% |
| Acta Parasitologica | 28% |
| Acta Zoologica | 62% |
| Acta Zoologica Fennica | Not available |
| Advanced Materials Research | Not available |
| Advances in Ecological Sciences | Not available |
| Advances in Marine Biology | 92% |
| Aerobiologia | 71% |
| African Entomology | 57% |
| African Invertebrates | 24% |
| African Journal of Herpetology | 56% |
| African Zoology | 48% |
| Afterall | 76% |
| Agricultural and Forest Meteorology | 97% |
| Agriculture, Ecosystems and Environment | 99% |
| AICCM Bulletin | 42% |
| Alcheringa | 57% |
| Alpine Botany | 79% |
| Alytes | Not available |
| Ambio | 97% |
| American Journal of Botany | 85% |
| American Journal of Physical Anthropology | 96% |
| American Journal of Science | Not available |
| American Malacological Bulletin | 37% |
| American Mineralogist | 82% |
| American Museum Novitates | 98% |
| American Naturalist | 90% |
| Amphibia-Reptilia | 72% |
| Analyst | 79% |
| Analytica Chimica Acta | 91% |
| Analytical Methods | 91% |
| Anatomical Record | Not available |
| Angewandte Chemie - International Edition | 97% |
| Animal Behaviour | 89% |
| Animal Biodiversity and Conservation | 53% |
| Animal Camouflage: Mechanisms and Function | Not available |
| Animal Conservation | 86% |
| Animal Evolution: Genomes, Fossils, and Trees | Not available |
| Animal Genetics | 90% |
| Annales de la Societe Entomologique de France | 51% |
| Annales de Limnologie | 37% |
| Annales Zoologici | 45% |
| Annals of Applied Biology | 78% |
| Annals of Botany | 95% |
| Annals of Science | Not available |
| Annals of the Entomological Society of America | 79% |
| Annals of the New York Academy of Sciences | 99% |
| Annual Review of Earth and Planetary Sciences | 99% |
| Annual Review of Ecology, Evolution, and Systematics | 99% |
| Annual Review of Entomology | 99% |
| Annual Review of Environment and Resources | 99% |
| Annual Review of Genomics and Human Genetics | 94% |
| Annual Review of Marine Science | 99% |
| Antarctic Science | 65% |
| Anthropocene Review | 90% |
| Anthropological Forum | 70% |
| Anthropology Today | 71% |
| Antiquity | 98% |
| Antonie van Leeuwenhoek, International Journal of General and Molecular Microbiology | 58% |
| ANZ Journal of Surgery | 48% |
| ANZIAM Journal | 54% |
| Apidologie | 80% |
| Applications in Plant Sciences | 70% |
| Applied and Environmental Microbiology | 91% |
| Applied and theoretical electrophoresis : the official journal of the International Electrophoresis Society | Not available |
| Applied Ecology and Human Dimensions in Biological Conservation | Not available |
| Applied Geochemistry | 78% |
| Applied Geography | 95% |
| Applied Physics Letters | 90% |
| Applied Spectroscopy | 83% |
| Aquaculture | 91% |
| Aquaculture Research | Not available |
| Aquatic Biology | 56% |
| Aquatic Biosystems | Not available |
| Aquatic Botany | Not available |
| Aquatic Conservation: Marine and Freshwater Ecosystems | Not available |
| Aquatic Ecosystem Health and Management | 48% |
| Aquatic Insects | 30% |
| Aquatic Invasions | 69% |
| Aquatic Living Resources | 47% |
| Aquatic Mammals | 55% |
| Aquatic Microbial Ecology | 67% |
| Aquatic Toxicology | 97% |
| Arabian Archaeology and Epigraphy | 95% |
| Arachnologische Mitteilungen | 36% |
| Arachnology | 17% |
| Archaeological and Anthropological Sciences | 93% |
| Archaeological Research at Caution Bay, Papua New Guinea: Cultural, Linguistic and Environmental Setting | Not available |
| Archaeologies | 80% |
| Archaeology in Oceania | 88% |
| Archaeometry | 97% |
| Archeological Papers of the American Anthropological Association | 72% |
| Archiv fur Hydrobiologie | Not available |
| Archiv fur Molluskenkunde | 41% |
| Archiv fur Protistenkunde | Not available |
| Archival Science | 95% |
| Archives and Manuscripts | 45% |
| Archives and Records | 91% |
| Archives of Natural History | 66% |
| Archives of Pharmacal Research | 81% |
| Arkivoc | 16% |
| Arthropod Structure and Development | 83% |
| Arthropod Systematics and Phylogeny | 76% |
| Arthropoda Selecta | 40% |
| Asian Myrmecology | 52% |
| Asian Perspectives | 91% |
| Astrobiology | 97% |
| Astronomy and Geophysics | 23% |
| Atlantic Geology | 44% |
| Atoll Research Bulletin | 37% |
| Auk | 91% |
| AusIMM Bulletin | Not available |
| Australian Journal of Marine and Freshwater Research | Not available |
| Austral Ark: The State of Wildlife in Australia and New Zealand | Not available |
| Austral Ecology | 64% |
| Austral Entomology | 70% |
| Australasian Corrosion Association Annual Conference: Corrosion and Prevention 2015, ACA 2015 | Not available |
| Australasian Historical Archaeology | 15% |
| Australasian Institute of Mining and Metallurgy Publication Series | Not available |
| Australasian Journal of Environmental Management | 73% |
| Australasian Plant Pathology | 60% |
| Australasian Science | Not available |
| Australian Aboriginal Studies | Not available |
| Australian Archaeology | 84% |
| Australian bird watcher | Not available |
| Australian Entomologist | Not available |
| Australian Feminist Studies | 58% |
| Australian Field Ornithology | Not available |
| Australian Forestry | 79% |
| Australian Geographer | 76% |
| Australian Geographical Studies | Not available |
| Australian Historical Studies | 79% |
| Australian Journal of Agricultural and Resource Economics | 79% |
| Australian Journal of Anthropology | 65% |
| Australian Journal of Biological Sciences | Not available |
| Australian Journal of Botany | 59% |
| Australian Journal of Chemistry | 45% |
| Australian Journal of Earth Sciences | 66% |
| Australian Journal of Ecology | Not available |
| Australian Journal of Entomology | Not available |
| Australian Journal of Environmental Education | 62% |
| Australian Journal of Forensic Sciences | 52% |
| Australian Journal of Linguistics | 77% |
| Australian Journal of Politics and History | 70% |
| Australian Journal of Zoology | 46% |
| Australian Journal of Zoology - Supplementary Series | Not available |
| Australian Mammalogy | 67% |
| Australian Planner | 54% |
| Australian Systematic Botany | 67% |
| Australian Veterinary Journal | 70% |
| Australian Zoological Reviews | Not available |
| Australian Zoologist | 53% |
| Austrobaileya | 12% |
| Avian Conservation and Ecology | 60% |
| Avian Pathology | 90% |
| Bats in the Anthropocene: Conservation of Bats in a Changing World | Not available |
| Battlefield Events: Landscape, Commemoration and Heritage | Not available |
| Beagle | Not available |
| Behavioral Ecology | 92% |
| Behavioral Ecology and Sociobiology | 91% |
| Behaviour | 63% |
| Bernissart Dinosaurs and Early Cretaceous Terrestrial Ecosystems | Not available |
| Beyond Memory: Silence and the Aesthetics of Remembrance | Not available |
| Biochemical Genetics | 59% |
| Biochemical Systematics and Ecology | 46% |
| Biodiversity | Not available |
| Biodiversity and Conservation | 86% |
| Biodiversity Data Journal | 52% |
| Biofouling | 82% |
| Biogeographia | 43% |
| Biogeosciences | 95% |
| Biogeosciences Discussions | Not available |
| Bioinformatics | 99% |
| BioInvasions Records | 59% |
| Biological Bulletin | 72% |
| Biological Conservation | 95% |
| Biological Control | 89% |
| Biological Invasions | 88% |
| Biological Journal of the Linnean Society | 71% |
| Biological Reviews | 99% |
| Biological Sampling in the Deep Sea | Not available |
| Biologist | 6% |
| Biology | Not available |
| Biology and Philosophy | 94% |
| Biology Letters | 89% |
| Biology of Butterflyfishes | Not available |
| Biology of Damselfishes | Not available |
| Bioorganic and Medicinal Chemistry | 82% |
| Bioorganic and Medicinal Chemistry Letters | 74% |
| BioScience | 97% |
| Biotropica | 71% |
| Bird Conservation International | 83% |
| Birds of eucalypt forests and woodlands: ecology, conservation, management | Not available |
| BMC Bioinformatics | 92% |
| BMC Biology | 97% |
| BMC Ecology | 70% |
| BMC Evolutionary Biology | 89% |
| BMC Genomics | 76% |
| BMC Microbiology | 66% |
| BMC Research Notes | 56% |
| BMR Journal of Australian Geology & Geophysics | Not available |
| Boletin de la Sociedad Geologica Mexicana | 42% |
| Bollettino della Societa Paleontologica Italiana | 63% |
| Botanica Marina | 65% |
| Botanical Journal of the Linnean Society | 78% |
| Brain, Behavior and Evolution | 60% |
| Bryozoan Studies 2004 - Proceedings of the 13th International Bryozoology Association Conference | Not available |
| Bulletin - Bureau of Mineral Resources, Geology & Geophysics, Australia | Not available |
| Bulletin - Museum National d'Histoire Naturelle Section C: Sciences de la Terre | Not available |
| Bulletin of Entomological Research | 72% |
| Bulletin of Marine Science | 67% |
| Bulletin of the American Museum of Natural History | 82% |
| Bulletin of the British Ornithologists' Club | 16% |
| Bulletin of the Geological Society of America | 92% |
| Bulletin of Volcanology | 62% |
| Cahiers de Biologie Marine | 34% |
| Cambridge Archaeological Journal | 97% |
| Canadian Entomologist | 62% |
| Canadian Journal of Chemistry | 48% |
| Canadian Journal of Earth Sciences | 57% |
| Canadian Journal of Zoology | 67% |
| Canadian Mineralogist | 35% |
| Cancer Genetics | 50% |
| Cetacean Paleobiology | Not available |
| CFS Courier Forschungsinstitut Senckenberg | Not available |
| Chelonian Conservation and Biology | 59% |
| Chemical and Pharmaceutical Bulletin | 51% |
| Chemical Geology | 92% |
| Chemie der Erde | 74% |
| Chemistry and Biodiversity | 60% |
| Chemistry and Biology | Not available |
| Chemistry of Materials | 97% |
| ChemistrySelect | 58% |
| Children, Childhood and Cultural Heritage | Not available |
| Chimia | 38% |
| Chinese Journal of Oceanology and Limnology | Not available |
| Chungara | 77% |
| Cladistics | 97% |
| Climate Change Management | 26% |
| Clinical and Experimental Allergy | 76% |
| Clinical Cancer Research | 95% |
| Clinical Toxicology | 63% |
| Cochrane Database of Systematic Reviews | 89% |
| Cold Spring Harbor Protocols | 53% |
| Coleopterists Bulletin | 17% |
| Collection of Czechoslovak Chemical Communications | Not available |
| Communications Biology | 85% |
| Community Ecology | 43% |
| Comparative Biochemistry and Physiology - A Molecular and Integrative Physiology | 83% |
| Comparative Biochemistry and Physiology - B Biochemistry and Molecular Biology | 83% |
| Comparative Biochemistry and Physiology - Part D: Genomics and Proteomics | 87% |
| Comparative Parasitology | 21% |
| Comptes Rendus de l'Academie des Sciences - Serie III | Not available |
| Computers and Geosciences | 86% |
| Conciliation on Colonial Frontiers: Conflict, Performance and Commemoration in Australia and the Pacific Rim | Not available |
| Condor | 87% |
| Connecting Inquiry and Professional Learning in Education: International Perspectives and Practical Solutions | Not available |
| Conservation and Management of Archaeological Sites | 90% |
| Conservation Biology | 96% |
| Conservation Biology: Voices from the Tropics | Not available |
| Conservation Genetics | 74% |
| Conservation Genetics Resources | 45% |
| Conservation Letters | 99% |
| Conservation Physiology | 74% |
| Considering Animals: Contemporary Studies in Human-Animal Relation | Not available |
| Contemporary Pacific | 53% |
| Continental Shelf Research | 85% |
| Contributions to Mineralogy and Petrology | 84% |
| Contributions to Zoology | 69% |
| Copeia | 60% |
| Coral Reefs | 93% |
| Corella | Not available |
| Corrosion and Conservation of Cultural Heritage Metallic Artefacts | Not available |
| Corrosion and Prevention 2018 | Not available |
| Corrosion and Prevention 2019 | Not available |
| Corrosion Science | 92% |
| Cretaceous Research | 82% |
| Critical Reviews in Plant Sciences | 96% |
| Crustacea and Arthropod Relationships | Not available |
| Crustaceana | 36% |
| Crustaceana Monographs | Not available |
| Crystal Growth and Design | 88% |
| Current Biology | 98% |
| Current Developments in Bioerosion | Not available |
| Current Herpetology | 28% |
| Current Microbiology | 42% |
| Current Opinion in Environmental Sustainability | 99% |
| Current Science | 65% |
| Current Zoology | 95% |
| Cybium | 36% |
| Cytogenetic and Genome Research | 34% |
| Czech Polar Reports | 47% |
| Database | 90% |
| Deep-Sea Research Part I: Oceanographic Research Papers | 86% |
| Deep-Sea Research Part II: Topical Studies in Oceanography | 93% |
| Desert Peoples: Archaeological Perspectives | Not available |
| Development Genes and Evolution | 30% |
| Developmental and Comparative Immunology | 77% |
| Developments in Earth and Environmental Sciences | Not available |
| Developments in Precambrian Geology | Not available |
| Developments in Sedimentology | Not available |
| Diabetes 1988: proceedings of the 13th Congress of the International Diabetes Federation. ICS800 | Not available |
| Dinosaur tracks and traces | Not available |
| Diptera Diversity: Status, Challenges and Tools | Not available |
| Diseases of Aquatic Organisms | 60% |
| Diversity | 70% |
| Diversity and Distributions | 93% |
| Doklady Earth Sciences | 39% |
| Early Popular Visual Culture | 59% |
| Earth and Environmental Science Transactions of the Royal Society of Edinburgh | 79% |
| Earth and Life: Global Biodiversity, Extinction Intervals and Biogeographic Perturbations Through Time | Not available |
| Earth and Planetary Science Letters | 98% |
| Earth and Space Science | 68% |
| Earth Sciences History | 41% |
| Earth Surface Processes and Landforms | 94% |
| Earth-Science Reviews | 99% |
| Earth's Future | 98% |
| Echinoderms in a Changing World - Proceedings of the 13th International Echinoderm Conference, IEC 2009 | Not available |
| Ecography | 97% |
| EcoHealth | 82% |
| Ecohydrology | 92% |
| Ecological Applications | 93% |
| Ecological Consequences of Climate Change: Mechanisms, Conservation, and Management | Not available |
| Ecological Entomology | 75% |
| Ecological Genetics and Genomics | 53% |
| Ecological Indicators | 94% |
| Ecological Informatics | 89% |
| Ecological Management and Restoration | 53% |
| Ecological Modelling | 76% |
| Ecological Monographs | 98% |
| Ecology | 94% |
| Ecology and Evolution | 76% |
| Ecology and Society | 93% |
| Ecology Letters | 98% |
| Ecology of Fishes on Coral Reefs | Not available |
| Economic Geology | 94% |
| Ecosphere | 85% |
| Ecotropica | Not available |
| Elements | 93% |
| eLife | 90% |
| EMA - Emergency Medicine Australasia | 66% |
| Emerging Topics in Life Sciences | 84% |
| Emotion, Affective Practices, and the Past in the Present | Not available |
| Emu | 69% |
| Encyclopedia of Animal Behavior | Not available |
| Encyclopedia of Biodiversity: Second Edition | Not available |
| Encyclopedia of Bioinformatics and Computational Biology: ABC of Bioinformatics | Not available |
| Encyclopedia of Caves | Not available |
| Encyclopedia of Earth Sciences Series | Not available |
| Encyclopedia of Ecology | Not available |
| Encyclopedia of Geology | Not available |
| Encyclopedia of Marine Mammals | Not available |
| Encyclopedia of Ocean Sciences | Not available |
| Encyclopedia of Ocean Sciences: Second Edition | Not available |
| Encyclopedia of Quaternary Science | Not available |
| Encyclopedia of Quaternary Science: Second Edition | Not available |
| Endangered Species Research | 83% |
| Entomologia Experimentalis et Applicata | 82% |
| Entomologica Scandinavica | Not available |
| Entomological News | 14% |
| Entomological Science | 66% |
| Environment and History | 88% |
| Environmental Biology of Fishes | 61% |
| Environmental Chemistry | Not available |
| Environmental DNA | Not available |
| Environmental Entomology | 74% |
| Environmental Evidence | 88% |
| Environmental History | 89% |
| Environmental Microbiology | 95% |
| Environmental Modelling and Software | 90% |
| Environmental Monitoring and Assessment | 72% |
| Environmental Pollution | 96% |
| Environmental Research | 91% |
| Environmental Science and Policy | 97% |
| Environmental Science and Technology | 94% |
| Epidemiology and Infection | 69% |
| Episodes | 75% |
| Estuaries and Coasts | 87% |
| Estuarine, Coastal and Shelf Science | 82% |
| Estudios Geologicos | 39% |
| Ethnobotany Research and Applications | 71% |
| Ethnomusicology: A Contemporary Reader, Volume II | Not available |
| Ethnos | 89% |
| Ethology | 71% |
| Ethology Ecology and Evolution | 58% |
| European Journal of Entomology | 59% |
| European Journal of Mineralogy | 52% |
| European Journal of Plant Pathology | 83% |
| European Journal of Taxonomy | 52% |
| European Perceptions of Terra Australis | Not available |
| Evolution | 88% |
| Evolution and Development | 63% |
| Evolution in Action: Case studies in Adaptive Radiation, Speciation and the Origin of Biodiversity | Not available |
| Evolutionary Applications | 93% |
| Evolutionary Biology | 77% |
| Evolutionary Systematics | 46% |
| Evolutionary trends | Not available |
| Expedition into Empire: Exploratory Journeys and the Making of the Modern World | Not available |
| Expeditionary Anthropology: Teamwork, Travel and the 'Science of Man' | Not available |
| Experimental and Applied Acarology | 71% |
| Experimental Parasitology | 51% |
| Exploration Geophysics | 47% |
| F1000Research | 81% |
| Facies | 75% |
| Far Eastern Entomologist | 37% |
| Fauna Norvegica | 24% |
| Fauna of Arabia | Not available |
| FEBS Journal | 84% |
| FEMS Microbiology Ecology | 90% |
| Fish and Fisheries | 99% |
| Fish and Shellfish Immunology | 95% |
| Fish Locomotion: An Eco-ethological Perspective | Not available |
| Fish Parasites: Pathobiology and Protection | Not available |
| Fisheries and Aquatic Sciences | 47% |
| Fisheries Research | 78% |
| Fisheries Science | 46% |
| Fishery Bulletin | 39% |
| Fishes Out of Water: Biology and Ecology of Mudskippers | Not available |
| Florida Entomologist | 47% |
| Folia Parasitologica | 43% |
| Folklore | 75% |
| Forensic Science International | 94% |
| Forensic Science International: Genetics | 93% |
| Forensic Science International: Genetics Supplement Series | 24% |
| Forensic Science, Medicine, and Pathology | 44% |
| Forest Ecology and Management | 91% |
| Forktail | Not available |
| Fossils and Strata | Not available |
| Freshwater Biology | 94% |
| Freshwater Science | 73% |
| Frontiers in Ecology and Evolution | 72% |
| Frontiers in Ecology and the Environment | 98% |
| Frontiers in Genetics | 44% |
| Frontiers in Marine Science | 89% |
| Frontiers in Microbiology | 84% |
| Frontiers in Physiology | 70% |
| Frontiers in Plant Science | 95% |
| Frontiers in Zoology | 94% |
| Frontiers of Biogeography | 39% |
| Fuel Processing Technology | 95% |
| Functional Ecology | 95% |
| Fundamental and Applied Nematology | Not available |
| Fungal Biology Reviews | 89% |
| Fungal Ecology | 87% |
| Gastrotricha and Gnathifera | Not available |
| Gems and Gemology | 23% |
| Gene | 73% |
| General and Comparative Endocrinology | 93% |
| General Anthropology | 26% |
| Genes | 55% |
| Genes and Genetic Systems | 61% |
| Genetica | 68% |
| Genetical Research | 23% |
| Genetics | 81% |
| Genome | 55% |
| Genome Biology | 98% |
| Genome Biology and Evolution | 89% |
| Genome Research | 98% |
| Geoarchaeology - An International Journal | 92% |
| Geobiology | 95% |
| Geobios | 60% |
| Geochemistry, Geophysics, Geosystems | 82% |
| Geochimica et Cosmochimica Acta | 94% |
| Geochronometria | 52% |
| Geodiversitas | 60% |
| Geographical Research | 81% |
| Geoheritage | 83% |
| Geologica Belgica | 53% |
| Geological Journal | 65% |
| Geological Magazine | 75% |
| Geological Society Memoir | 95% |
| Geological Society Special Publication | 90% |
| Geologie en Mijnbouw/Netherlands Journal of Geosciences | 86% |
| Geology | 97% |
| Geology Today | 37% |
| Geomicrobiology Journal | 75% |
| Geophysics | 89% |
| Geosciences (Switzerland) | 76% |
| Geothermics | 93% |
| GFF | 59% |
| GigaScience | 97% |
| Global and Planetary Change | 96% |
| Global Change Biology | 98% |
| Global Ecology and Biogeography | 96% |
| Global Ecology and Conservation | 69% |
| Global Environmental Change | 99% |
| Global Masculinities and Manhood | Not available |
| Going Forward by Looking Back: Archaeological Perspectives on Socio-Ecological Crisis, Response, and Collapse | Not available |
| Gondwana Research | 99% |
| Gulf of Mexico Origin, Waters, and Biota | Not available |
| Handbook of Australasian Biogeography | Not available |
| Handbook of Environmental Chemistry, Volume 5: Water Pollution | Not available |
| Handbook of Forensic Anthropology and Archaeology | Not available |
| Handbook of Landscape Archaeology | Not available |
| Handbook of Postcolonial Archaeology | Not available |
| Handbook of Research on Development and Religion | Not available |
| Handbook of Zoology: Anthropoda: Insecta | Not available |
| Helgoland Marine Research | 68% |
| Hereditas | 36% |
| Heredity | 70% |
| Heritage | 70% |
| Heritage Science | 94% |
| Herpetofauna | Not available |
| Herpetologica | 65% |
| Herpetological Bulletin | 27% |
| Herpetological Conservation and Biology | 45% |
| Herpetological Journal | 47% |
| Herpetological Monographs | 87% |
| Herpetological Review | 21% |
| Herpetology Notes | 25% |
| Heterocycles | 26% |
| Himalayan Geology | 26% |
| Historical Biology | 84% |
| Historical Records of Australian Science | 53% |
| History and Anthropology | 93% |
| History Australia | 26% |
| Holocene | 96% |
| Human and Ecological Risk Assessment | 85% |
| Human Dimensions of Wildlife | 68% |
| Human Ecology | 91% |
| Humanities for the Environment: Integrating Knowledge, Forging New Constellations of Practice | Not available |
| Hunting the Gatherers: Ethnographic Collectors, Agents, and Agency in Melanesia 1870s-1930s | Not available |
| Hydrobiologia | 88% |
| Hydrogeology Journal | 82% |
| Hydrological Sciences Journal | 79% |
| Hydrology and Earth System Sciences | 97% |
| Hydrometallurgy | 90% |
| Hyperfine Interactions | 35% |
| Hystrix | 69% |
| Ibis | 85% |
| ICES Journal of Marine Science | 92% |
| ICHIM07 - International Cultural Heritage Informatics Meeting, Proceedings | Not available |
| Ichthyological Exploration of Freshwaters | 75% |
| Ichthyological Research | 43% |
| IFIP Advances in Information and Communication Technology | 26% |
| IMA Fungus | 97% |
| Imitation and Social Learning in Robots, Humans and Animals: Behavioural, Social and Communicative Dimensions | Not available |
| Immunogenetics | 61% |
| Indian Journal of Fisheries | 16% |
| Indian Journal of Geo-Marine Sciences | 17% |
| Indian Journal of Marine Sciences | Not available |
| Indigenous Peoples and Demography: The Complex Relation between Identity and Statistics | Not available |
| Infection, Genetics and Evolution | 86% |
| Inland Waters | 66% |
| Inorganic Chemistry | 89% |
| Insect Conservation and Diversity | 89% |
| Insect Molecular Biology | 90% |
| Insect Science | 93% |
| Insect Systematics and Diversity | 23% |
| Insect Systematics and Evolution | 72% |
| Insectes Sociaux | 69% |
| Insects | 61% |
| Integrative and Comparative Biology | 93% |
| Integrative Zoology | 88% |
| Integrity and Historical Research | Not available |
| Interdisciplinary Contributions to Archaeology | Not available |
| Interface Focus | 90% |
| Internal Medicine Journal | 44% |
| International Journal for Parasitology | 90% |
| International Journal for Parasitology: Parasites and Wildlife | 76% |
| International Journal of Acarology | 50% |
| International Journal of Applied Earth Observation and Geoinformation | 99% |
| International Journal of Arts and Technology | 84% |
| International Journal of Biological Macromolecules | 87% |
| International Journal of Climatology | 89% |
| International Journal of Cultural Property | 81% |
| International Journal of Developmental Biology | 40% |
| International Journal of Earth Sciences | 85% |
| International Journal of Heritage Studies | 98% |
| International Journal of Historical Archaeology | 90% |
| International Journal of Legal Medicine | 74% |
| International Journal of Molecular Sciences | 83% |
| International Journal of Nautical Archaeology | 94% |
| International Journal of Odonatology | 49% |
| International Journal of Osteoarchaeology | 88% |
| International Journal of Plant Sciences | 62% |
| International Journal of Radiation Applications and Instrumentation. Part B, Nuclear medicine and biology | Not available |
| International Journal of Sustainable Development and World Ecology | 93% |
| International Journal of the History of Sport | 64% |
| International Journal of the Inclusive Museum | 52% |
| International Journal of Tourism Research | 89% |
| International Journal of Wildland Fire | 91% |
| International Journal of Zoology | 41% |
| International Sugar Journal | 8% |
| Invertebrate Biology | 52% |
| Invertebrate Reproduction and Development | 45% |
| Invertebrate Systematics | 81% |
| Invertebrate Taxonomy | Not available |
| Invertebrate Zoology | 53% |
| Investigative Genetics | Not available |
| iScience | 88% |
| ISME Journal | 98% |
| Israel Journal of Entomology | 13% |
| Japanese Journal of Ichthyology | Not available |
| Journal - Royal Society of Western Australia | 55% |
| Journal and Proceedings of the Royal Society of New South Wales | 14% |
| Journal de la Societe des Oceanistes | 60% |
| Journal for Nature Conservation | 74% |
| Journal of African Earth Sciences | 73% |
| Journal of Anatomy | 74% |
| Journal of Animal Ecology | 96% |
| Journal of Anthropological Archaeology | 98% |
| Journal of Apicultural Research | 86% |
| Journal of Applied Crystallography | 81% |
| Journal of Applied Ecology | 95% |
| Journal of Applied Entomology | 82% |
| Journal of Applied Ichthyology | 35% |
| Journal of Aquatic Animal Health | 62% |
| Journal of Arabic Literature | 82% |
| Journal of Arachnology | 60% |
| Journal of Archaeological Method and Theory | 99% |
| Journal of Archaeological Research | 99% |
| Journal of Archaeological Science | 98% |
| Journal of Archaeological Science: Reports | 91% |
| Journal of Arid Environments | 71% |
| Journal of Asia-Pacific Biodiversity | 50% |
| Journal of Asia-Pacific Entomology | 59% |
| Journal of Asian Earth Sciences | 90% |
| Journal of Australian Studies | 97% |
| Journal of Avian Biology | 83% |
| Journal of Avian Medicine and Surgery | 32% |
| Journal of Biogeography | 91% |
| Journal of Biological Research (Greece) | 73% |
| Journal of Biosciences | 69% |
| Journal of Cave and Karst Studies | 42% |
| Journal of Cetacean Research and Management | 35% |
| Journal of Chemical Ecology | 76% |
| Journal of Clinical Forensic Medicine | Not available |
| Journal of Crustacean Biology | 47% |
| Journal of Cultural Heritage | 99% |
| Journal of Economic Entomology | 80% |
| Journal of Ecotourism | 86% |
| Journal of Electroanalytical Chemistry | 84% |
| Journal of Environmental Assessment Policy and Management | 53% |
| Journal of Environmental Management | 95% |
| Journal of Ethnobiology | 85% |
| Journal of Ethology | 56% |
| Journal of Eukaryotic Microbiology | 63% |
| Journal of Evolutionary Biology | 83% |
| Journal of Experimental Biology | 90% |
| Journal of Experimental Marine Biology and Ecology | 84% |
| Journal of Experimental Zoology Part A: Ecological and Integrative Physiology | 79% |
| Journal of Experimental Zoology Part B: Molecular and Developmental Evolution | 82% |
| Journal of Field Archaeology | 92% |
| Journal of Field Ornithology | 64% |
| Journal of Fish Biology | 59% |
| Journal of Fish Diseases | 81% |
| Journal of Forensic Sciences | 59% |
| Journal of Geochemical Exploration | 93% |
| Journal of Geophysical Research: Solid Earth | 91% |
| Journal of Geosciences | Not available |
| Journal of Geosciences (Czech Republic) | 63% |
| Journal of Global Responsibility | Not available |
| Journal of Great Lakes Research | 70% |
| Journal of Helminthology | 79% |
| Journal of Heredity | 68% |
| Journal of Herpetology | 65% |
| Journal of Human Evolution | 98% |
| Journal of Hymenoptera Research | 64% |
| Journal of Iberian Geology | 64% |
| Journal of Immunology | 78% |
| Journal of Insect Biodiversity | 14% |
| Journal of Insect Conservation | 75% |
| Journal of Insect Physiology | 86% |
| Journal of Insect Science | 63% |
| Journal of Interdisciplinary History | 95% |
| Journal of Invertebrate Pathology | 80% |
| Journal of Island and Coastal Archaeology | 99% |
| Journal of Linguistics | 91% |
| Journal of Magnetism and Magnetic Materials | 76% |
| Journal of Mammalian Evolution | 82% |
| Journal of Mammalogy | 81% |
| Journal of Maps | 87% |
| Journal of Marine Science and Technology (Taiwan) | 66% |
| Journal of Marine Systems | 85% |
| Journal of Maritime Archaeology | 76% |
| Journal of Materials Chemistry | Not available |
| Journal of Medicinal Chemistry | 99% |
| Journal of Metamorphic Geology | 95% |
| Journal of Microbiological Methods | 53% |
| Journal of Micropalaeontology | 76% |
| Journal of Microscopy | 69% |
| Journal of Mineralogical and Petrological Sciences | 43% |
| Journal of Molecular Evolution | 69% |
| Journal of Molecular Structure | 70% |
| Journal of Molluscan Studies | 65% |
| Journal of Morphology | 70% |
| Journal of Natural History | 42% |
| Journal of Natural Products | 94% |
| Journal of Nematology | 55% |
| Journal of Ocean University of China | 43% |
| Journal of Organic Chemistry | 87% |
| Journal of Ornithology | 74% |
| Journal of Paediatrics and Child Health | 59% |
| Journal of Palaeogeography | 79% |
| Journal of Paleontology | 71% |
| Journal of Parasitology | 43% |
| Journal of Pest Science | 96% |
| Journal of Petrology | 88% |
| Journal of Plankton Research | 76% |
| Journal of Proteome Research | 84% |
| Journal of Proteomics | 88% |
| Journal of Quaternary Science | 95% |
| Journal of Radioanalytical and Nuclear Chemistry | 58% |
| Journal of Raman Spectroscopy | 69% |
| Journal of Raptor Research | 57% |
| Journal of Reproduction and Fertility | Not available |
| Journal of Research Practice | 87% |
| Journal of Sea Research | 73% |
| Journal of Shellfish Research | 40% |
| Journal of Solid State Chemistry | 75% |
| Journal of Solution Chemistry | 35% |
| Journal of South American Earth Sciences | 61% |
| Journal of Structural Biology | 57% |
| Journal of Structural Geology | 83% |
| Journal of Submicroscopic Cytology and Pathology | Not available |
| Journal of Sulfur Chemistry | 64% |
| Journal of Synchrotron Radiation | 80% |
| Journal of Systematic Palaeontology | 93% |
| Journal of Systematics and Evolution | 91% |
| Journal of the American Ceramic Society | 85% |
| Journal of the American Chemical Society | 98% |
| Journal of the American Institute for Conservation | 90% |
| Journal of the Anthropological Society of South Australia | 60% |
| Journal of the Botanical Research Institute of Texas | 18% |
| Journal of the Entomological Research Society | 16% |
| Journal of the Geological Society | 84% |
| Journal of the Geological Society of Australia | Not available |
| Journal of the History of Biology | 79% |
| Journal of the Institute of Conservation | 85% |
| Journal of the International Association for Mathematical Geology | Not available |
| Journal of the Lepidopterists' Society | 28% |
| Journal of the Malacological Society of Australia | Not available |
| Journal of the Marine Biological Association of the United Kingdom | 51% |
| Journal of the Mechanical Behavior of Biomedical Materials | 86% |
| Journal of the New York Entomological Society | Not available |
| Journal of the North American Benthological Society | Not available |
| Journal of the Royal Society Interface | 87% |
| Journal of the Royal Society of New Zealand | 79% |
| Journal of the Royal Society of Western Australia | 28% |
| Journal of Theoretical Biology | 91% |
| Journal of Thermal Analysis and Calorimetry | 77% |
| Journal of Thermal Biology | 85% |
| Journal of Threatened Taxa | 31% |
| Journal of Toxicology - Clinical Toxicology | Not available |
| Journal of Travel Medicine | 98% |
| Journal of Tropical Ecology | 56% |
| Journal of Urban Ecology | 81% |
| Journal of Vegetation Science | 83% |
| Journal of Vertebrate Paleontology | 69% |
| Journal of Virological Methods | 39% |
| Journal of Volcanology and Geothermal Research | 79% |
| Journal of Wildlife Diseases | 58% |
| Journal of Wildlife Management | 77% |
| Journal of Zoological Systematics and Evolutionary Research | 78% |
| Journal of Zoology | 80% |
| Kanunnah | Not available |
| Kimberlites II: The Mantle and Crust-Mantle Relationships | Not available |
| Kukila | 26% |
| Landscape and Urban Planning | 99% |
| Landscape Ecology | 93% |
| Lankesteriana | 36% |
| Laser Cleaning II | Not available |
| Laterality | 67% |
| Lecture Notes in Computer Science (including subseries Lecture Notes in Artificial Intelligence and Lecture Notes in Bioinformatics) | 50% |
| Lecture Notes in Earth System Sciences | 29% |
| Lethaia | 85% |
| Library Review | Not available |
| Lichenologist | 60% |
| Life in the World's Oceans: Diversity, Distribution, and Abundance | Not available |
| Limnologica | 74% |
| Limnology and Oceanography | 97% |
| Limnology and Oceanography: Methods | 82% |
| Lithic Technology | 81% |
| Lithos | 88% |
| Living in a Dynamic Tropical Forest Landscape | Not available |
| Living Under the Shadow: Cultural Impacts of Volcanic Eruptions | Not available |
| Malacologia | 97% |
| Mammal Research | 64% |
| Mammal Review | 97% |
| Mammalia | 54% |
| Mammalian Biology | 77% |
| Mammalian Genome | 65% |
| Management of Biological Invasions | 81% |
| Marine and Freshwater Behaviour and Physiology | 35% |
| Marine and Freshwater Research | 72% |
| Marine Biodiversity | 66% |
| Marine Biodiversity Records | 54% |
| Marine Biology | 79% |
| Marine Biology Research | 60% |
| Marine Biotechnology | 91% |
| Marine Drugs | 86% |
| Marine Ecology | 65% |
| Marine Ecology Progress Series | 81% |
| Marine Environmental Research | 98% |
| Marine Genomics | 61% |
| Marine Mammal Science | 73% |
| Marine Ornithology | 29% |
| Marine Policy | 97% |
| Marine Pollution Bulletin | 97% |
| Marsupial Genetics and Genomics | Not available |
| Marsupials | Not available |
| Materials and Corrosion | 71% |
| Medical and Veterinary Entomology | 95% |
| Medical Journal of Australia | 88% |
| Mediterranean Marine Science | 72% |
| Memoirs - Association of Australasian Palaeontologists | Not available |
| Memoir of the Geological Society of America | 59% |
| Memoires - Geological Society of India | Not available |
| Memoirs of Museum Victoria | 64% |
| Memoirs of the Association of Australasian Palaeontologists | Not available |
| Memoirs of the Entomological Society of Canada | Not available |
| Memoirs of the Queensland Museum | 21% |
| Memoirs of the Queensland Museum: Cultural Heritage Series | Not available |
| Metabarcoding and Metagenomics | 95% |
| Meteoritics | Not available |
| Meteoritics and Planetary Science | 66% |
| Methods in Ecology and Evolution | 97% |
| MethodsX | 46% |
| Microbial Ecology | 93% |
| Microscopy and Microanalysis | 55% |
| Microscopy Research and Technique |  |
| Migrant, Multicultural and Diasporic Heritage: Beyond and Between Borders | Not available |
| Mineral Processing and Extractive Metallurgy Review | 75% |
| Mineralium Deposita | 96% |
| Mineralogical Magazine | 48% |
| Mineralogical Record | Not available |
| Mineralogy and Petrology | 62% |
| Minerals | 69% |
| Minerals Engineering | 90% |
| Mitochondrial DNA | Not available |
| Mitochondrial DNA Part A: DNA Mapping, Sequencing, and Analysis | 32% |
| Mitochondrial DNA Part B: Resources | 32% |
| Mobile DNA | 47% |
| Modern Geology | Not available |
| Molecular and Biochemical Parasitology | 54% |
| Molecular and Cellular Probes | 24% |
| Molecular and Cellular Proteomics | 90% |
| Molecular Biology and Evolution | 99% |
| Molecular Biology Reports | 18% |
| Molecular Ecology | 96% |
| Molecular Ecology Notes | Not available |
| Molecular Ecology Resources | 97% |
| Molecular Immunology | 65% |
| Molecular Microbiology | 77% |
| Molecular Phylogenetics and Evolution | 93% |
| Molecules | 74% |
| Molluscan Research | 49% |
| Monthly Notices of the Royal Astronomical Society | 87% |
| Morphology and Systematics (Elateroidea, Bostrichiformia, Cucujiformia partim) | Not available |
| Muelleria | Not available |
| Museum | 12% |
| Museum Anthropology | 91% |
| Museum Communication and Social Media: The Connected Museum | Not available |
| Museum History Journal | 58% |
| Museum International | 47% |
| Museum Management and Curatorship | 95% |
| Museum Management and Marketing | Not available |
| Museum Materialities: Objects, Engagements, Interpretations | Not available |
| Museum Revolutions: How Museums Change and are Changed | Not available |
| Museum Worlds | 31% |
| Museums and their Communities | Not available |
| Museums, Sexuality, and Gender Activism | Not available |
| Museums, Society, Inequality | Not available |
| MycoKeys | 72% |
| Mycologia | 74% |
| Mycological Research | Not available |
| Myrmecological News | 95% |
| National Geographic Research | Not available |
| National Geographic Research & Exploration | Not available |
| National Identities | 91% |
| Natura Croatica | 26% |
| Natural Disasters and Cultural Change | Not available |
| Natural Product Communications | 53% |
| Natural Product Letters | Not available |
| Natural Product Research | 74% |
| Nature | 99% |
| Nature Climate Change | 99% |
| Nature Communications | 97% |
| Nature conservation: the role of remnants of native vegetation | Not available |
| Nature Ecology and Evolution | 99% |
| Nature Genetics | 99% |
| Nature Geoscience | 99% |
| Nature, Temporality and Environmental Management: Scandinavian and Australian perspectives on peoples and landscapes | Not available |
| Naturwissenschaften | 70% |
| Nautilus | 12% |
| Nematologica | Not available |
| NeoBiota | 94% |
| Neues Jahrbuch fur Geologie und Palaontologie - Abhandlungen | 42% |
| Neues Jahrbuch fur Mineralogie, Abhandlungen | 27% |
| Neues Jahrbuch fur Mineralogie, Monatshefte | Not available |
| New Heritage: New Media and Cultural Heritage | Not available |
| New Phytologist | 98% |
| New Scientist | 6% |
| New Zealand Entomologist | 21% |
| New Zealand Journal of Botany | 52% |
| New Zealand Journal of Ecology | 64% |
| New Zealand Journal of Geology and Geophysics | 62% |
| New Zealand Journal of History | Not available |
| New Zealand Journal of Marine and Freshwater Research | 68% |
| New Zealand Journal of Zoology | 58% |
| New Zealand Plant Protection | 46% |
| Nieuwelaan 38 | Not available |
| NIWA Biodiversity Memoirs | Not available |
| Norsk Geologisk Tidsskrift | 70% |
| North-Western Journal of Zoology | 28% |
| Northern Territory Naturalist | Not available |
| Notornis | 50% |
| Nutrients | 90% |
| Obsidian and Ancient Manufactured Glasses | Not available |
| Ocean and Coastal Management | 91% |
| Oceania | 82% |
| Oceanography and Marine Biology | 98% |
| Odonatologica | 32% |
| Oecologia | 83% |
| Oikos | 88% |
| On the Ecology of Australia's Arid Zone | Not available |
| One Earth | 80% |
| Open Biology | 86% |
| Open Geosciences | 51% |
| Ophelia | Not available |
| Oral History and Public Memories | Not available |
| Orbit (London) | 44% |
| Ore Geology Reviews | 82% |
| Organic Geochemistry | 87% |
| Organic Letters | 92% |
| Organisms Diversity and Evolution | 72% |
| Oriental Insects | 35% |
| Oryx | 79% |
| Ostrich | 48% |
| Pacific Conservation Biology | 58% |
| Pacific Rim congress 87. Proc. international congress, 1987. Gold Coast, Queensland | Not available |
| Pacific Science | 67% |
| Palaeontologische Zeitschrift | Not available |
| Palaeobiodiversity and Palaeoenvironments | 69% |
| Palaeobiology II | Not available |
| Palaeogeography and Palaeobiogeography: Biodiversity in Space and Time | Not available |
| Palaeogeography, Palaeoclimatology, Palaeoecology | 94% |
| Palaeontographica Canadiana | Not available |
| Palaeontographica, Abteilung A: Palaozoologie - Stratigraphie | 88% |
| Palaeontologia Electronica | 58% |
| Palaeontology | 98% |
| Palaeoworld | 79% |
| Palaios | 79% |
| Palaontologische Zeitschrift | 55% |
| Paleobiology | 82% |
| Paleontological Research | 33% |
| Paleontology in Ecology and Conservation | Not available |
| Palynology | 62% |
| Pan-Pacific Entomologist | 11% |
| Papeis Avulsos de Zoologia | 37% |
| Papers and Proceedings of the Royal Society of Tasmania | 17% |
| Papers in Palaeontology | 86% |
| Parasite | 86% |
| Parasites and Vectors | 82% |
| Parasitology | 93% |
| Parasitology International | 57% |
| Parasitology Research | 87% |
| Parasitology Today | Not available |
| Pathogens | 40% |
| PeerJ | 83% |
| People and nature conservation | Not available |
| Peptides | 68% |
| Perspectives in Plant Ecology, Evolution and Systematics | 86% |
| Philosophical Transactions of the Royal Society B: Biological Sciences | 96% |
| Phuket Marine Biological Center Research Bulletin | 23% |
| Phycologia | 79% |
| Phyllomedusa | 28% |
| Phylogenetic Diversity: Applications and Challenges in Biodiversity Science | Not available |
| Physica B: Condensed Matter | 67% |
| Physics and Chemistry of Minerals | 49% |
| Physics Education | 44% |
| Physiological and Biochemical Zoology | 89% |
| Phytopathology | 88% |
| Phytotaxa | 50% |
| Plankton and Benthos Research | 35% |
| Plant and Fungal Systematics | 54% |
| Plant Biology | 87% |
| Plant Conservation Science and Practice: The Role of Botanic Gardens | Not available |
| Plant Ecology | 67% |
| Plant Protection Quarterly | Not available |
| Plant Systematics and Evolution | 68% |
| Planta Medica | 88% |
| Pleistoannelida, Sedentaria III and Errantia I | Not available |
| PLoS Biology | 97% |
| PLoS Genetics | 95% |
| PLoS Neglected Tropical Diseases | 93% |
| PLoS ONE | 92% |
| Polar Biology | 81% |
| Polar Record | 60% |
| Polar Science | 66% |
| Polish Polar Research | 60% |
| Pollination Services to Agriculture: Sustaining and Enhancing a Key Ecosystem Service | Not available |
| Possums and gliders | Not available |
| Powder Diffraction | 34% |
| Precambrian Research | 93% |
| Prehled Vyzkumu | 67% |
| Proc. 1st international polychaete conference, Sydney, 1983 | Not available |
| Proceedings - 2017 International Conference on Data Science and Advanced Analytics, DSAA 2017 | Not available |
| Proceedings - 21st International Congress on Modelling and Simulation, MODSIM 2015 | Not available |
| Proceedings - Ecological Society of Australia | Not available |
| Proceedings - IEEE 15th International Conference on eScience, eScience 2019 | Not available |
| Proceedings - Royal Society of Victoria | Not available |
| Proceedings of SPIE - The International Society for Optical Engineering | Not available |
| Proceedings of the 2018 3rd Digital Heritage International Congress, Digital Heritage 2018 - Held jointly with the 2018 24th International Conference on Virtual Systems and Multimedia, VSMM 2018 | Not available |
| Proceedings of the Academy of Natural Sciences of Philadelphia | 47% |
| Proceedings of the Biological Society of Washington | 38% |
| Proceedings of the Entomological Society of Washington | 31% |
| Proceedings of the first international polychaete conference, Sydney, Australia, July 1983. | Not available |
| Proceedings of the Geologists' Association | 63% |
| Proceedings of the International Astronautical Congress, IAC | 11% |
| Proceedings of the International Conference on Information Visualisation | 14% |
| Proceedings of the Linnean Society of New South Wales | Not available |
| Proceedings of the National Academy of Sciences of the United States of America | 95% |
| Proceedings of the Prehistoric Society | Not available |
| Proceedings of the Royal Society B: Biological Sciences | 92% |
| Proceedings of the Royal Society of Queensland | 44% |
| Proceedings of the Royal Society of Victoria | 32% |
| Proceedings. Biological sciences | 92% |
| Progress in Natural Science | 77% |
| Proteins: Structure, Function and Bioinformatics | 63% |
| Protist | 49% |
| Psyche (London) | Not available |
| Psyche (New York) | Not available |
| Psychiatry, Psychology and Law | 71% |
| Public Historian | 74% |
| Publications of the Astronomical Society of Australia | 92% |
| QJM - Monthly Journal of the Association of Physicians | 78% |
| Quaternary International | 82% |
| Quaternary Research | 84% |
| Quaternary Science Reviews | 99% |
| Queensland Archaeological Research | 84% |
| Queensland Geographical Journal | Not available |
| Queensland Review | 56% |
| Queering the Museum | Not available |
| Race, Empire and First World War Writing | Not available |
| Radiation Physics and Chemistry | 72% |
| Radiocarbon | 96% |
| Raffles Bulletin of Zoology | 38% |
| Reaction Kinetics and Catalysis Letters | Not available |
| ReCollections | Not available |
| Reconstructing the Tree of Life: Taxonomy and Systematics of Species Rich Taxa | Not available |
| Records of the Australian Museum | 85% |
| Regional Studies in Marine Science | 51% |
| Regulated Rivers: Research & Management | Not available |
| Regulatory Peptides | Not available |
| Reintroduction of Top-Order Predators | Not available |
| Remote Sensing in Ecology and Conservation | 94% |
| Report - International Whaling Commission | Not available |
| Report - International Whaling Commission, Special Issue | Not available |
| Reproduction | 90% |
| Reproduction, Fertility and Development | 86% |
| Resource Geology | 67% |
| Restaurator | 66% |
| Restoration Ecology | 84% |
| Rethinking Invasion Ecologies from the Environmental Humanities | Not available |
| Review of Palaeobotany and Palynology | 67% |
| Review of Scientific Instruments | 57% |
| Reviews in Fish Biology and Fisheries | 98% |
| Revista de Biologia Tropical | 44% |
| Revue Suisse de Zoologie | 28% |
| River Conservation and Management | Not available |
| Rocks and Minerals | 9% |
| Rodents: Habitat, Pathology and Environmental Impact | Not available |
| Royal Society Open Science | 91% |
| RSC Advances | 82% |
| Russian Journal of Herpetology | 22% |
| Salamandra | 73% |
| Schweizerische Mineralogische und Petrographische Mitteilungen | Not available |
| Science | 98% |
| Science Advances | 97% |
| Science in New Guinea | Not available |
| Science of the Total Environment | 96% |
| Science Progress | 53% |
| Scientia Marina | 46% |
| Scientific American | 25% |
| Scientific Data | 98% |
| Scientific Reports | 93% |
| Scientific Tourism: Researchers as Travellers | Not available |
| Scottish Journal of Geology | 42% |
| Scripta Geologica | Not available |
| Search | Not available |
| Seas at the millennium - an environmental evaluation - Volume 2 | Not available |
| Sedimentary Geology | 82% |
| Sedimentology | 89% |
| Senckenbergiana Lethaea | Not available |
| Senckenbergiana Maritima | Not available |
| Separations | 52% |
| Society of Petroleum Engineers - SPE International Conference and Exhibition on Health, Safety, Security, Environment, and Social Responsibility 2018 | Not available |
| Sociobiology | 38% |
| Soil Biology and Biochemistry | 98% |
| Soils Stones and Symbols: Cultural Perceptions of the Mineral World | Not available |
| Solid State Ionics | 82% |
| South African Journal of Geology | 54% |
| South African Journal of Marine Science | Not available |
| South African Journal of Science | 69% |
| South Asian Survey | Not available |
| South Australian Geographical Journal | Not available |
| South Australian Ornithologist | Not available |
| Southwestern Naturalist | 9% |
| Special Paper of the Geological Society of America | 48% |
| Special Papers in Palaeontology | Not available |
| Species Diversity | 26% |
| Spectrochimica Acta - Part A: Molecular and Biomolecular Spectroscopy | 93% |
| Spixiana | 31% |
| Starfish: Biology and Ecology of the Asteroidea | Starfish: Biology and Ecology of the Asteroidea |
| STEM Education in Primary Classrooms: Unravelling Contemporary Approaches in Australia and New Zealand | Not available |
| Studies in Conservation | 86% |
| Studies in the History of Gardens and Designed Landscapes | 48% |
| Subterranean Biology | 70% |
| Sunbird | Not available |
| Symbiosis | 80% |
| Systematic and Applied Acarology | 70% |
| Systematic Biology | 98% |
| Systematic Entomology | 97% |
| Systematic Parasitology | 34% |
| Systematics and Biodiversity | 74% |
| Talanta | 91% |
| Tasforests | Not available |
| Taxon | 80% |
| Teaching and Teacher Education | 96% |
| Telopea | 10% |
| Terra Nova | 80% |
| Terrible Hard Biscuits: A reader in Aboriginal History | Not available |
| Tetrahedron | 63% |
| Tetrahedron Letters | 58% |
| Thalassas | 18% |
| The Anatomical Record | 72% |
| The Archaeology and Anthropology of Landscape: Shaping Your Landscape | Not available |
| The Archaeology of Australia's Deserts | Not available |
| The Archaeology of Watercraft Abandonment | Not available |
| The Australian Art Field: Practices, Policies, Institutions | Not available |
| The Biogeography of the Australian North West Shelf: Environmental Change and Life's Response | Not available |
| The biology of Australasian frogs and reptiles | Not available |
| The Biology of Lungfishes | Not available |
| The Bioregional Imagination: Literature, Ecology, and Place | Not available |
| The British Journal for the History of Science | 90% |
| The Broken Promise of Agricultural Progress: An Environmental History | Not available |
| The Canadian Entomologist | 62% |
| The Care and Conservation of Geological Material: Minerals, Rocks, Meteorites and Lunar Finds | Not available |
| The Evolution of Plant Physiology | Not available |
| The Future of Nature: Documents of Global Change | Not available |
| The Geologic Time Scale 2012 | Not available |
| The Handbook of Plant Biosecurity: Principles and Practices for the Identification, Containment and Control of Organisms that Threaten Agriculture and the Environment Globally | Not available |
| The International Journal of Nautical Archaeology | 94% |
| The Journal of parasitology | 43% |
| The Journal of Protozoology | Not available |
| The Long Way Home: The Meaning and Values of Repatriation | Not available |
| The Mekong | Not available |
| The Oxford Handbook of Maritime Archaeology | Not available |
| The Prehistory of Food: Appetites for Change | Not available |
| The Routledge Companion to Indigenous Repatriation: Return, Reconcile, Renew | Not available |
| The Routledge Handbook of Philosophy of Biodiversity | Not available |
| Theoretical and Applied Climatology | 70% |
| Thermochimica Acta | 83% |
| Tijdschrift voor Entomologie | 33% |
| Tissue and Cell | 36% |
| Tourism in Marine Environments | 54% |
| Tourism Management | 99% |
| Toxicon | 39% |
| Toxins | 75% |
| Transactions - Geological Society of South Africa | Not available |
| Transactions - Geothermal Resources Council | 21% |
| Transactions of the Institutions of Mining and Metallurgy, Section C: Mineral Processing and Extractive Metallurgy | Not available |
| Transactions of the Royal Society of Edinburgh: Earth Sciences | Not available |
| Transactions of the Royal Society of South Africa | 66% |
| Transforming Practice: Selections from the Journal of Museum Education, 1992-1999 | Not available |
| Treetops at Risk: Challenges of Global Canopy Ecology and Conservation | Not available |
| Trends in Ecology and Evolution | 99% |
| Trends in Parasitology | 97% |
| Tropical Biomedicine | 23% |
| Tropical Medicine and Infectious Disease | 68% |
| Tropical Natural History | 30% |
| Tropical Zoology | 34% |
| Turkish Journal of Veterinary and Animal Sciences | 41% |
| Turkish Journal of Zoology | 42% |
| Uncertain Images: Museums and the Work of Photographs | Not available |
| Urban Ecosystems | 91% |
| Vector-Borne and Zoonotic Diseases | 58% |
| Vertebrate Paleobiology and Paleoanthropology | 83% |
| Vertebrate Zoology | 47% |
| Veterinaria Italiana | 65% |
| Veterinary Microbiology | 98% |
| Veterinary Parasitology | 5% |
| Veterinary Record | 41% |
| Vibrational Spectroscopy | 49% |
| Victorian Naturalist | 11% |
| Vie et Milieu | 32% |
| Viruses | 73% |
| Visitor Studies | 93% |
| Was Man More Aquatic in the Past? Fifty Years After Alister Hardy - Waterside Hypotheses of Human Evolution | Not available |
| Waterbirds | 38% |
| Wellbeing and Place | Not available |
| Wetlands Ecology and Management | 57% |
| Wildlife Biology | 54% |
| Wildlife Forensic Investigation: Principles and Practice | Not available |
| Wildlife Forensics: Methods and Applications | Not available |
| Wildlife Research | 63% |
| Wiley Interdisciplinary Reviews: Climate Change | 99% |
| Wombats | Not available |
| World Archaeology | 94% |
| World Seas: An Environmental Evaluation Volume II: The Indian Ocean to the Pacific | Not available |
| X-Ray Spectrometry | 25% |
| Yale Journal of Biology and Medicine | 82% |
| Zeitschrift fur Kristallographie | 9% |
| Zoo Biology | 57% |
| ZooKeys | 63% |
| Zoologica Scripta | 95% |
| Zoological Journal of the Linnean Society | 94% |
| Zoological Letters | 83% |
| Zoological Research | 92% |
| Zoological Science | 42% |
| Zoological Studies | 66% |
| Zoologischer Anzeiger | 71% |
| Zoology | 77% |
| Zoology in the Middle East | 40% |
| Zoomorphology | 60% |
| Zoos' Print Journal | Not available |
| Zoosystema | 59% |
| Zoosystematics and Evolution | 67% |
| Zootaxa | 54% |
