# Supplementary material for: Research publications of Australia’s natural history museums, 1981–2020: Enduring relevance in a changing world
Source: PLoS One. 2023 Jun 23;18(6):e0287659. doi: 10.1371/journal.pone.0287659 (PMC10289469; doi:10.1371/journal.pone.0287659)
Supplement: S5 Table — All pairwise comparisons differ significantly (p = 0.032–0.008). Shading from red to green denotes increasing value for the R-statistic. (DOCX) [file pone.0287659.s005.docx]

**S5 Table. *R*-statistic values from a one-way ANOSIM test on the sources of documents published by authors from the ANHMs in various year groups. All pairwise comparisons differ significantly (*p* = 0.032 – 0.008). Shading from red to green denotes increasing value for the *R*-statistic.**

|  | **1981-85** | **1986-90** | **1991-95** | **1996-20** | **2001-05** | **2006-10** | **2011-15** |
| --- | --- | --- | --- | --- | --- | --- | --- |
| **1986-90** | 0.632 |  |  |  |  |  |  |
| **1991-95** | 0.984 | 0.448 |  |  |  |  |  |
| **1996-20** | 0.992 | 0.964 | 0.992 |  |  |  |  |
| **2001-05** | 1.000 | 1.000 | 0.996 | 0.680 |  |  |  |
| **2006-10** | 1.000 | 1.000 | 1.000 | 1.000 | 0.628 |  |  |
| **2011-15** | 1.000 | 1.000 | 1.000 | 1.000 | 0.980 | 0.508 |  |
| **2016-20** | 1.000 | 1.000 | 1.000 | 1.000 | 0.992 | 0.944 | 0.680 |
