# Supplementary material for: Research publications of Australia’s natural history museums, 1981–2020: Enduring relevance in a changing world
Source: PLoS One. 2023 Jun 23;18(6):e0287659. doi: 10.1371/journal.pone.0287659 (PMC10289469; doi:10.1371/journal.pone.0287659)
Supplement: S1 File — (DOCX) [file pone.0287659.s006.docx]

**Supporting Information S1 Methods**

In more detail, we chose Scopus for our analysis of the publications of the ANHMs because Scopus:

- Has a comprehensive institutional affiliation feature, assigning documents to institutional affiliations given by authors. In comparing Scopus and WoS, Donner and colleagues [5] found that Scopus recognised a wider range of institutions. From their check list of 445 German institutions WoS retrieved only 20%, while Scopus retrieved 85%. They did note, though, that Scopus often employed fine-scale disambiguation that separated for example, hospitals within universities from the parent universities. For the current study, all eight ANHMs had an affiliation ID in Scopus, whereas only five were listed by affiliation in WoSCC.
- Covers the period 1981-2020. Initially, Scopus mainly included recent reference material (since 1996), but this has now been extended back to at least 1970, and older material is being added regularly [6].
- Lists a range of research outputs (sources in Scopus language) likely to cover museum research outputs, including serial publications with an ISSN (e.g., journals, book or conference series) and other publications that have an ISBN (e.g., books, book chapters, stand-alone conferences) [6]. Initially, books were included only when the book was part of a named series. From 2013, Scopus began including a much wider range of books and book chapters, going back to 2003 for the Social Sciences, Arts and Humanities, and 2005 for Science, Technology and Medicine [7].
- Provides a wide range of bibliometric data.
- Has output that could be replicated by anyone with a Scopus subscription following specified search strings.

Scopus refers to all records in the database as documents, which is the term we use to refer collectively to all items identified in searches.

Custom search strings of unique museum affiliation identification numbers were used to return documents in which one or more authors claimed an affiliation with any ANHM for documents authored between 1981 and 2020 inclusive. Multiple museum affiliation identification numbers exist for the Museum and Art Gallery of the Northern Territory, and several documents published in affiliation with this ANHM are published under the synonym of the Northern Territory Museum of Arts and Sciences. Similarly, publications from state museums in Victoria may be grouped under an ID for Museum Victoria or Museums Victoria and the Queensland Museum has a separate affiliation for the Museum of Tropical Queensland in Townsville (Supporting Information S2 Table).

Document data were downloaded in .csv formatted files, which were retained as master files. File copies were created in Microsoft Excel for sorting and data manipulation, allowing return at any time to the uncorrupted master files if errors were made in manipulating records. We searched for duplicate records and removed them before analysis, summing the citations for the two records into the one retained. Duplicates arose mainly because of journal name changes, where identical documents appeared under two journal names for several years before or after the change. We located these duplicates and removed the one with the incorrect title for the year of publication, summing the citations between the pairs to give a correct total for the document. There were also several strange cases where some documents appeared twice in the same journal and issue, with different page numbers in each entry. We located the original documents in all cases to confirm a duplicate and removed the version with the incorrect page numbers, summing citations to the version retained. The documents for the Tasmanian Museum and Art Gallery included some documents from the Tasmanian Herbarium, which is included under the same Scopus Affiliation ID. We checked these documents and removed those where the authors indicated affiliation to the Herbarium specifically, because herbaria data were not included in the searches for the other ANHMs. The final cleaned data file is included as Supporting Information S3 table.

The final table includes no retracted papers. There were only 12 Erratum notices, spread over 9 years. We did not exclude them, considering this number trivial in relation to the overall size of the dataset.

**References**

1. Calver MC, Goldman B, Hutchings PA, Kingsford RT. Why discrepancies in searching the conservation biology literature matter. Biol Conserv. 2017;213:19-26. doi: 10.1016/j.biocon.2017.06.028.

2. Calver MC, Crawford HM, Fletcher D. A century of peer-reviewed Australian zoological research: Prominent authors, themes and usage of papers from *Australian Zoologist*. Aust Zool. 2021;41(4):663-88. doi: 10.7882/AZ.2021.024.

3. Harzing A-W, Alakangas S. Google Scholar, Scopus and the Web of Science: a longitudinal and cross-disciplinary comparison. Scientometrics. 2016;106(2):787-804. doi: 10.1007/s11192-015-1798-9.

4. Meho LI, Yang K. Impact of data sources on citation counts and rankings of LIS faculty: Web of science versus scopus and google scholar. J Assoc Inf Sci Technol 2007;58(13):2105-25.

5. Donner P, Rimmert C, van Eck NJ. Comparing institutional-level bibliometric research performance indicator values based on different affiliation disambiguation systems. Quant Sci Stud. 2020;1(1):150-70. doi: 10.1162/qss_a_00013.

6. Elsevier. Scopus Content Coverage Guide 2020 [cited 2022 8th April]. Available from: https://www.elsevier.com/__data/assets/pdf_file/0007/69451/Scopus_ContentCoverage_Guide_WEB.pdf.

7. Elsevier. Scopus book expansion project nearly complete 2015 [cited 2022 8th April]. Available from: https://blog.scopus.com/posts/scopus-book-expansion-project-nearly-complete.
