# Supplementary figures and images for: Research publications of Australia’s natural history museums, 1981–2020: Enduring relevance in a changing world
Source: PLoS One. 2023 Jun 23;18(6):e0287659. doi: 10.1371/journal.pone.0287659 (PMC10289469; doi:10.1371/journal.pone.0287659)

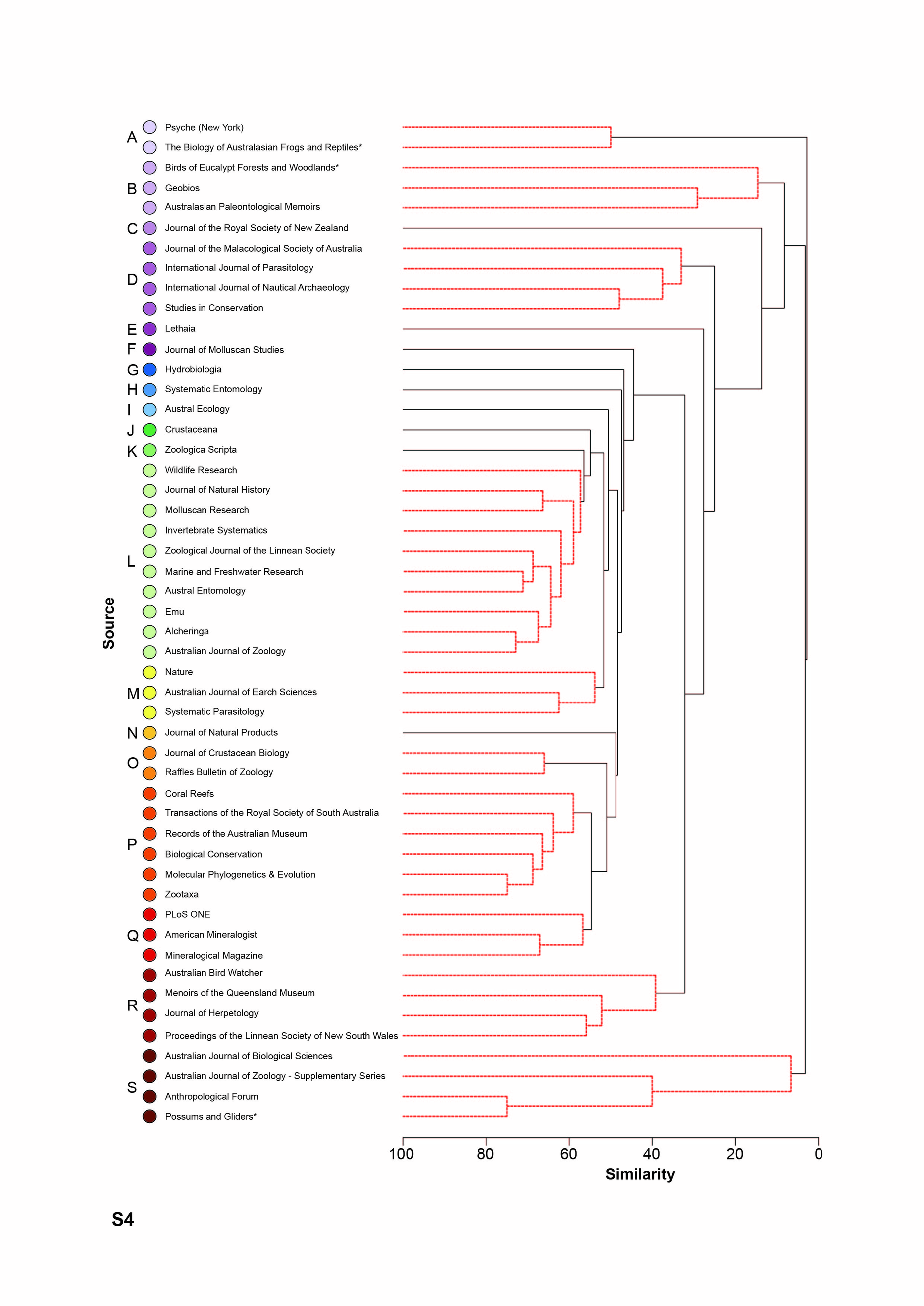

Supplement: S1 Fig — The clusters to the left of each black vertical line represent sources whose temporal pattern of publication were shown by SIMPROF not to be significantly different from each other (p > 0.05), but to be significantly different from those in all other groups of samples (p < 0.05). * denotes sources that are edited books rather than journals. (TIF) [file pone.0287659.s007.tif]

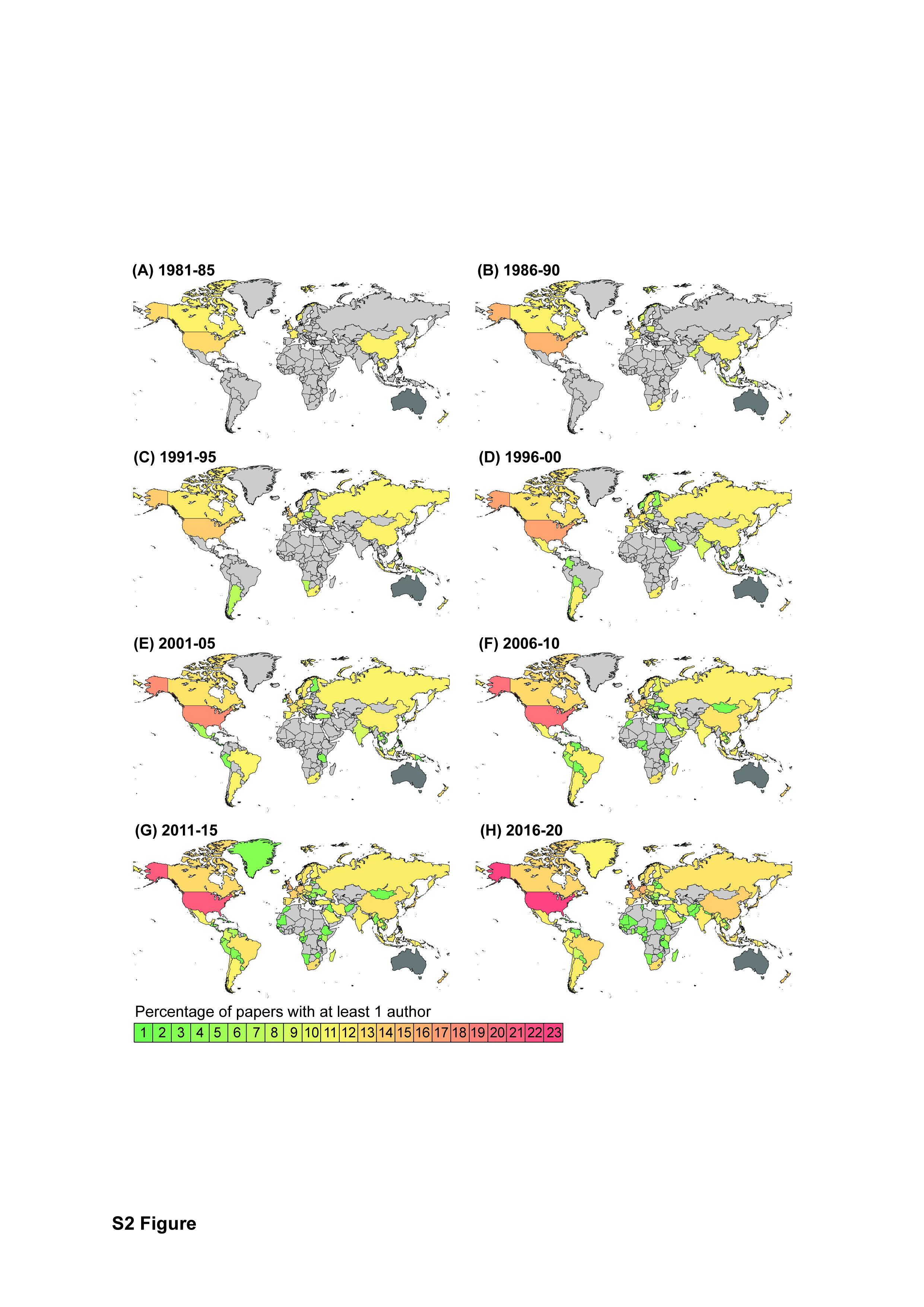

Supplement: S2 Fig — ANHMs are based in Australia, so there is at least one Australian author on all these documents. Therefore, Australia is shown in dark grey to indicate that Australian authors are excluded. The map was produced using MapChart software’s free version licence https://www.mapchart.net/terms.html#licensing-maps, under a CC BY license with permission of Minas Giannekas, founder and developer of MapChart. (TIF) [file pone.0287659.s008.tif]

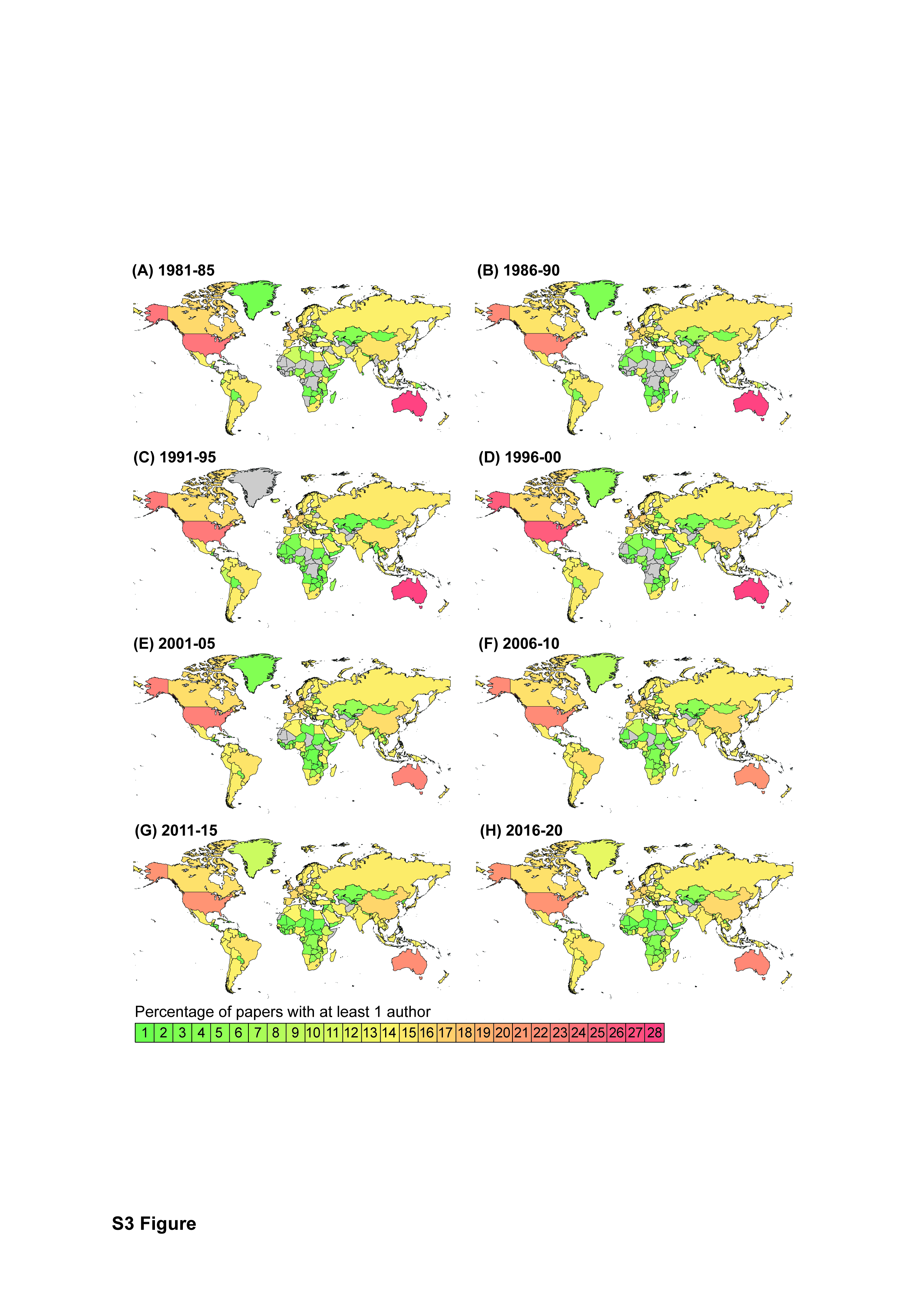

Supplement: S3 Fig — The map was produced using MapChart software’s free version licence https://www.mapchart.net/terms.html#licensing-maps, under a CC BY license with permission of Minas Giannekas, founder and developer of MapChart. (TIF) [file pone.0287659.s009.tif]
